# Redox-Controlled Proton Gating in Bovine Cytochrome *c* Oxidase

**DOI:** 10.1371/journal.pone.0063669

**Published:** 2013-05-16

**Authors:** Tsuyoshi Egawa, Syun-Ru Yeh, Denis L. Rousseau

**Affiliations:** Department of Physiology and Biophysics, Albert Einstein College of Medicine, Bronx, New York, United States of America; Instituto Tecnologia Quimica e Biologica; Universidade Nova de Lisboa, Portugal

## Abstract

Cytochrome *c* oxidase is the terminal enzyme in the electron transfer chain of essentially all organisms that utilize oxygen to generate energy. It reduces oxygen to water and harnesses the energy to pump protons across the mitochondrial membrane in eukaryotes and the plasma membrane in prokaryotes. The mechanism by which proton pumping is coupled to the oxygen reduction reaction remains unresolved, owing to the difficulty of visualizing proton movement within the massive membrane-associated protein matrix. Here, with a novel hydrogen/deuterium exchange resonance Raman spectroscopy method, we have identified two critical elements of the proton pump: a proton loading site near the propionate groups of heme *a*, which is capable of transiently storing protons uploaded from the negative-side of the membrane prior to their release into the positive side of the membrane and a conformational gate that controls proton translocation in response to the change in the redox state of heme *a*. These findings form the basis for a postulated molecular model describing a detailed mechanism by which unidirectional proton translocation is coupled to electron transfer from heme *a* to heme *a*_3_, associated with the oxygen chemistry occurring in the heme *a*_3_ site, during enzymatic turnover.

## Introduction

Cytochrome *c* oxidase (C*c*O) is the terminal enzyme in the electron transfer chain in both eukaryotic and prokaryotic cells. It catalyzes the four-electron reduction of dioxygen to water at the catalytic site formed by a binuclear center, consisting of a high-spin heme (heme *a*_3_) and a copper atom (Cu_B_). In addition to the heme-copper binuclear center, the enzyme has a low-spin heme (heme *a*), which mediates electron transfer from cytochrome *c* (cyt *c*) to the binuclear center [1], [2]. C*c*O harnesses the energy released from the oxygen reduction chemistry to pump protons across the inner membrane of mitochondria in eukaryotic cells or the plasma membrane in prokaryotic cells, thereby contributing to the proton motive force utilized by ATP synthase for the synthesis of ATP. Despite its importance and an enormous amount of research over several decades [3]–[7], the mechanism by which proton pumping is coupled to the oxygen reduction reaction remains unresolved.

**Figure 1:**
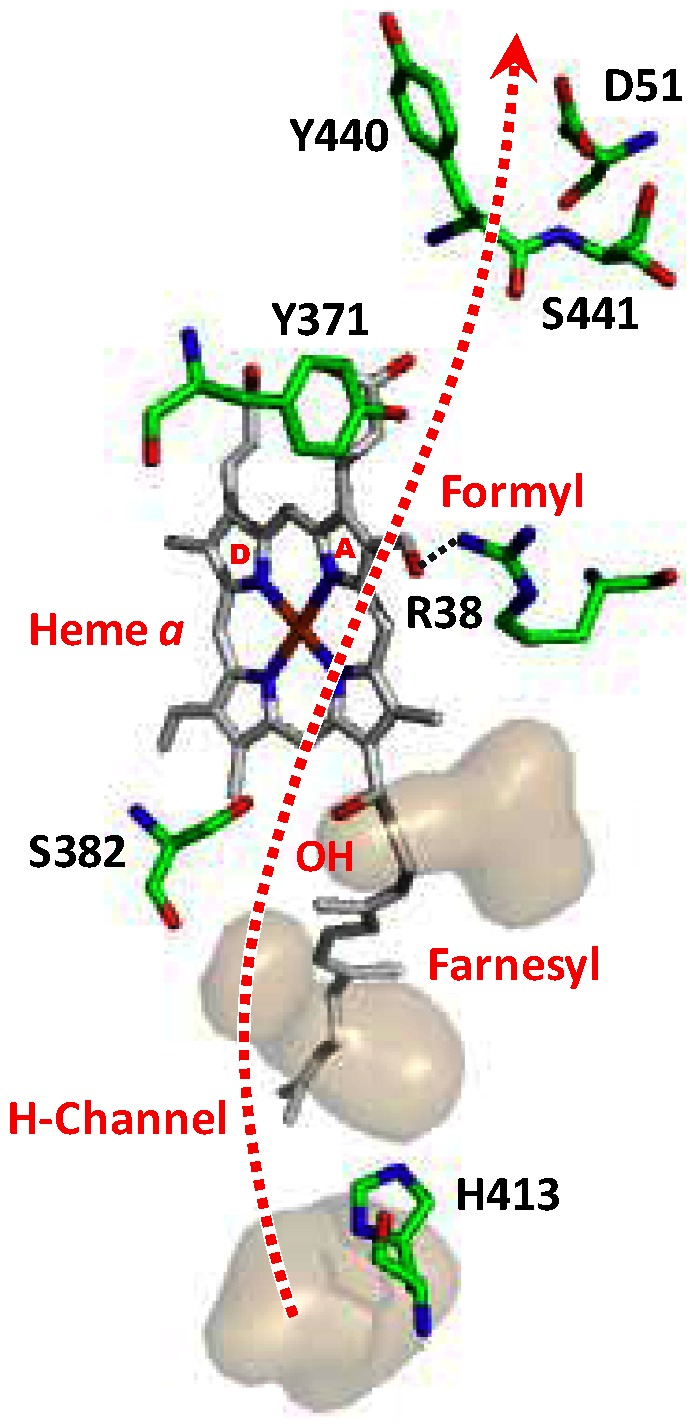
Postulated H-Channel for proton translocation in bC*c*O. The structure was drawn with the PyMol Molecular Graphics System, Schrodinger, LLC. The large voids found near the H-Chanel in the crystal structure of oxidized bC*c*O (PDB: 3ABL), where water may be stored, are highlighted by the gray surfaces generated by the software “Hollow” [47] with a 1.3 Å sphere.

During each catalytic cycle of C*c*O, four electrons, provided by cyt *c*, and four protons (the “chemical” protons), taken up from the negative side (**n**-side) of the mitochondrial membrane (or the cytoplasmic membrane), are used to reduce dioxygen to water; at the same time four protons (the “pumped” protons) are pumped across the membrane from the **n**-side to the positive side (**p**-side) of the mitochondrial intermembrane space (or the periplasmic space) [1], [2]. Site-directed mutagenesis [8], [9] and X-ray crystallographic studies of mammalian and bacterial oxidases have identified three potential proton conduction pathways, the H, D, and K-channels (named after conserved amino acid residues on the **n**-side of the postulated pathways) [10]–[12]. The functional importance of the three channels in proton translocation, as well as in providing chemical protons for the dioxygen reduction chemistry, has been extensively studied [7], [13], [14]. Evidence from studies of bacterial C*c*Os has shown that the K-channel delivers chemical protons from the **n**-side of the membrane to the binuclear center, while the D-channel provides both the chemical protons and the pumped protons. It is thought that the D-channel conducts protons from the **n**-side surface to E242 (bovine C*c*O numbering) near heme *a*_3_, where chemical protons are directed to the binuclear center, whereas the pumped protons are routed to a postulated proton loading site prior to their release to the **p**-side surface [2], [15], [16]. In contrast, it has been proposed that, in mammalian C*c*O, pumped protons pass through the H-channel starting from the **n**-side surface and terminate at D51 on the **p**-side surface, mediated by redox-dependent changes in water cavities residing between the **n**-side of the membrane and the heme *a* macrocycle (Figure 1) [17]. (See the File S1 for details.) Support for translocation via the H-channel in mammalian C*c*O has been supplied by site-directed mutagenesis studies in HeLa cells by Shimada and co-workers [18], [19]. Several years prior to the identification of the H-channel, proton-electron coupling at heme *a* was proposed by Konstantinov and coworkers [20], [21], who identified a redox Bohr effect associated with heme *a*. The redox Bohr effect was subsequently reported by others as well [22], supporting a role of heme *a* in mediating proton translocation [23], [24]. Moreover, spectroscopic results, reported by Babcock and Callahan also supported the scenario that heme *a* plays a central role in proton pumping [25], [26]. The existence of distinct channels and mechanisms for proton translocation in mammalian and bacterial enzymes is controversial as it challenges our common perception that the general features of the proton pumping mechanism are conserved in the C*c*O family of enzymes [21], [27]. So far, no consensus has been reached owing to the difficulty of visualizing proton movement within the massive matrix of the integral membrane protein during the oxygen chemistry. To clarify this issue it is essential to determine the translocation mechanisms of the mammalian and the bacterial enzymes at the atomic detail level.

Recently, with resonance Raman spectroscopy, we discovered that the CH_2_ twisting and wagging vibrational modes of each of the four propionate groups associated with heme *a* and *a*_3_ display significant solvent H/D isotope sensitivity in the reduced derivative of both the bovine and bacterial enzymes [28], [29]. As there are no exchangeable protons on the CH_2_ groups of the propionates, the isotope sensitivities of these vibrational modes are ascribed to exchangeable protons residing on the carboxyl groups of the propionates and/or the amino acids/water molecules that form H-bonds with them. Our studies showed that the vibrational modes associated with each of the four propionates can be differentiated [29], as each propionate adapts a distinct conformation due to its unique surrounding environment. In this work, we took advantage of the site-specificity of these H/D sensitive propionate vibrational modes by using them as markers for proton accessibility to the two active heme centers in bovine C*c*O (bC*c*O). With this novel H/D exchange resonance Raman spectroscopic method, we found that the H/D exchange near the heme *a* propionates is allowed when heme *a* is oxidized, but is inhibited when heme *a* is reduced. In addition, there is no proton leakage between the regions near the propionates of heme *a* and the propionates of heme *a*_3_. On the basis of these discoveries, a molecular model is postulated to describe the mechanism by which unidirectional proton translocation near heme *a* is coupled to the oxygen chemistry occurring at the binuclear center in bC*c*O.

## Materials and Methods

The bC*c*O protein was isolated as described by Yoshikawa and coworkers [30]. The protein samples were purified in a buffer containing 0.1 M sodium phosphate, pH 7.4, with 0.1% n-decyl-β-maltoside and stored at −80°C until the H/D exchange treatments. The purified resting protein had Soret and visible bands at 423 and 598 nm, respectively, characteristic of the “fast” enzyme [31]. The deuterated buffers were prepared using high purity D_2_O (99 atom % D) from Cambridge Isotope Laboratories, Inc. (Andover, MA). The D_2_O buffer contained 0.1% n-decyl-β-maltoside and 0.1 M Tris, pD 8.5. The H_2_O buffers used (pH 8.5) had the same concentrations of n-decyl-β-maltoside and Tris. The pD of the D_2_O buffer was measured using an ordinary pH meter assuming that the reading of the meter was lower by 0.39 as compared to the pD value. To prepare the fully H to D exchanged samples, the stock bC*c*O solution was diluted into the D_2_O buffer at a 1∶9 ratio, and the diluted solution was concentrated to ∼10% volume using a Millipore concentrator. Repeating this process three times enabled ∼99 atom % D (98.9% in theory) in the sample preparations. To reduce the samples, a slight excess of sodium dithionite was used. To assure that artifacts were not produced by the dithionite, the experiments were repeated using a large excess of ascorbate (50 mM) as the reductant and cytochrome *c* (10 µM) as a mediator for ∼30 µM (after the dilution) bC*c*O, which was the standard bC*c*O concentration in our resonance Raman measurements.

The **P_M_** species in these studies was generated by the CO/O_2_ incubation method [32]. In the H/D exchange experiments on **P_M_**, we continuously recorded the visible-absorption spectrum immediately after the dilution until the initiation of reduction to assure that the sample was always in the **P_M_** form during the incubation period. Based on an analysis of the extinction coefficients of the **P** species [33] and the oxidized enzyme, the population of the **P_M_** form during the incubation period was determined to be >87%.

Resonance Raman scattering, from samples in a spinning cell, was excited by the 441.6 nm line of a He-Cd laser (Kimmon Electric, Centennial, CO), and dispersed by a Spex 1.25 m polychromator equipped with a charge-coupled device detector (Princeton Instrument, 1100 PB). The laser power at the sample cell was 10 mW for the fully-reduced form and 1 mW for the reduced-CO and MV-SH̄ forms. Optical absorption spectra were measured on a Shimadzu UV2100U spectrophotometer. For the resonance Raman measurements, the dilution experiments were designed so that the final concentration of the bC*c*O samples for each heme was 30 µM. The resonance Raman intensity from the samples at the same concentration was sometimes slightly different, depending on small changes of the laser power. Therefore, for the spectral difference calculations, each resonance Raman spectrum was normalized on the basis of the ν_4_ band intensity, which is the strongest band among the porphyrin core vibrations.

## Results

**Figure 2:**
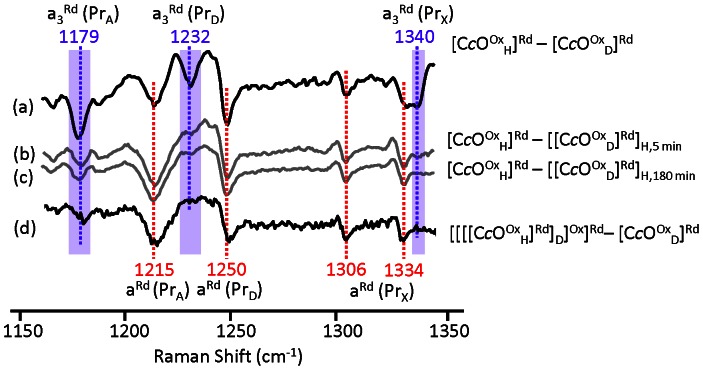
H/D exchange resonance Raman difference spectra of the fully reduced and turn-over forms of bC*c*O. The vibrational modes associated with the ring A and D propionate groups of heme *a*_3_ and heme *a* are indicated; the subscript “X” stands for the ring A and/or D propionate groups, as a specific assignment could not be made. The reference difference spectrum ([C*c*O^Ox^_H_]^Rd^–[C*c*O^Ox^_D_]^Rd^) is shown in (a) in which the oxidized enzyme was incubated in either H_2_O ([C*c*O^Ox^_H_]) or D_2_O ([C*c*O^Ox^_D_]) for over 8 hours prior to reduction and spectral acquisition. In difference spectra (b) and (c) the fully deuterated sample was reduced and exposed to protonated buffer for either 5 or 180 minutes, respectively, prior to spectral acquisition. In difference spectra (d) a deuterated reduced sample was placed in protonated buffer, turned over by exposure to O_2_ and then reduced for the spectral acquisition.

To detect H/D sensitive propionate modes of the fully reduced bC*c*O, two samples, [C*c*O^Ox^_H_]^Rd^ and [C*c*O^Ox^_D_]^Rd^, were prepared. Here, the Ox-H-Rd or Ox-D-Rd in the superscript or subscript describes the sequence of sample treatment, starting from incubating a fully oxidized enzyme (superscript Ox) in a H_2_O (H) or D_2_O (D) buffer for ∼8 hours at 4°C, followed by reduction with sodium dithionite under a nitrogen atmosphere (superscript Rd). This nomenclature system is described in detail in the File S1 and used hereafter. Figure S1 shows the raw resonance Raman spectra of [C*c*O^Ox^_H_]^Rd^ and [C*c*O^Ox^_D_]^Rd^. The [C*c*O^Ox^_H_]^Rd^–[C*c*O^Ox^_D_]^Rd^ difference spectrum, calculated from the raw spectra, is shown in Figure 2 (Spectrum a). Incubation of the enzyme with H_2_O or D_2_O buffer for >8 hours resulted in a very similar difference spectrum, indicating that the H/D exchange had reached completion within 8 hours.

In the [C*c*O^Ox^_H_]^Rd^–[C*c*O^Ox^_D_]^Rd^ difference spectrum, several negative bands, such as those at 1179, 1215, 1232, 1250, 1306, 1334 and 1340 cm^−1^, are evident, indicating that the vibrational modes become narrower in D_2_O as compared to H_2_O. These bands have been assigned to vibrational modes associated with the propionate (Pr) groups of heme *a* and *a*_3_, on the basis of studies carried out with synthetic porphyrins [34], myoglobin with modified heme [34], [35], bacterial C*c*O mutants and CO-bound adducts [29]. Based on these results, the bands at 1179/1232 and 1215/1250 cm^−1^ are assigned to CH_2_ twisting modes of the Pr_A_/Pr_D_ sidechains attached to the A/D pyrrole rings of hemes *a*_3_ and *a*, respectively [29]. On the other hand, the bands at 1340 and 1306/1334 cm^−1^ are assigned to the CH_2_ wagging modes of the propionates of heme *a*_3_ and heme *a*, respectively [34], [35]. They are indicated as “Pr_X_”, as the modes associated with Pr_A_ and Pr_D_ could not be differentiated. In addition, some of the modes might involve Raman intensity contributions from both hemes, such as the line at 1179 cm^−1^ (See Figure S2). It is important to note that these H/D sensitive vibrational modes are readily detectable for the fully reduced enzyme, but not the fully oxidized enzyme.

### H/D Exchange in the Fully-reduced Enzyme

In bC*c*O, heme *a* and *a*_3_ are embedded in sites that are deep into the protein matrix, (∼ 1/3 down into the membrane from the **p**-side [36]). To examine the proton accessibility of the two heme sites from the bulk solvent, a fully oxidized bC*c*O sample was first incubated with D_2_O buffer (pH 8.5) at 4°C for ∼8 hours prior to its reduction to the reduced state; the reduced deuterated sample thus produced, [C*c*O^Ox^_D_]^Rd^, was then diluted into H_2_O buffer (by a 1∶9 ratio) and aged for a period of time, **t**, to allow for the H/D exchange to occur prior to spectral acquisition. For clarity, the sample is denoted as [[C*c*O^Ox^_D_]^Rd^]_H,t_, in which the superscripted/subscripted Ox-D-Rd-H indicate the order of the sample treatment as described above. The spectrum of a fully reduced sample in H_2_O, [C*c*O^Ox^_H_]^Rd^, was obtained as a reference. The [C*c*O^Ox^_H_]^Rd^ − [[C*c*O^Ox^_D_]^Rd^]_H,t_ difference spectrum was calculated and compared to Spectrum a in Figure 2. If the H/D exchange reaction does not occur at all at time **t**, the difference spectrum is expected to be similar to Spectrum a; on the other hand, if the H/D exchange reaction reaches completion, the difference spectrum is expected to appear as a flat featureless baseline (see File S1 for details).

As shown in Figure 2, at t = 5 min, nearly all the heme *a*_3_ associated peaks in the difference spectrum disappeared (only the 1179 cm^−1^ peak retained a small residual intensity). The data indicate that nearly complete H/D exchange occurred near the heme *a*_3_ propionates. In contrast, all the peaks associated with heme *a* remained present (although the *a*-Pr_D_ peak at 1250 cm^−1^ is slightly weakened). The data indicate that nearly no H/D exchange took place near the heme *a* propionates. The difference spectrum obtained at t = 180 min shows virtually identical features, indicating that the H/D exchange at the heme *a* site was fully inhibited in the reduced enzyme.

As a control, an equivalent set of experiments was carried out by diluting the reduced protonated sample, [C*c*O^Ox^_H_]^Rd^, in the D_2_O buffer (Spectra b and c in Figure S3). Similar conclusions were derived from the data, confirming that complete H/D exchange at the heme *a*_3_ site occurred within 5 min, whereas the H/D exchange at the heme *a* site was severely restricted. It is important to note that the complete exchange near heme *a*_3_ and the absence of exchange near heme *a* indicates that there is no H/D exchange crosstalk between the two heme sites (*i.e.* the H/D exchange taking place in the heme *a* site is independent of that in the heme *a*_3_ site). The observed absence H/D exchange at the reduced heme *a* was fully reproducible and not due to side effects caused by sodium dithionite, as the same results were observed when we used the more physiological reduction system for bC*c*O, ascorbic acid and cytochrome *c* (Figure S4).

### H/D Exchange in the Fully Oxidized Enzyme

**Figure 3:**
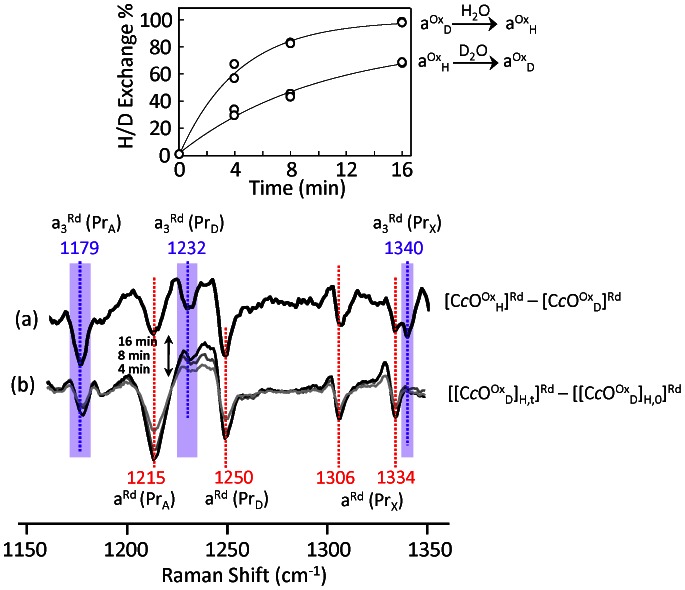
H/D exchange resonance Raman difference spectra of the fully oxidized bC*c*O. The [C*c*O^Ox^_H_]^Rd^–[C*c*O^Ox^_D_]^Rd^ spectrum is shown in (a) as a reference. The increase in the magnitude of the difference spectrum from 4 to 8 to 16 minutes is shown in (b). The top panel shows the degree of H/D exchange near the heme *a* site extracted from spectra b (indicated as a^ox^_D_→a^ox^_H_) as a function of time. As a comparison, the data associated with the equivalent reaction (a^ox^_H_→a^ox^_D_) obtained from the data shown in Figure S5 is also plotted.

To determine the H/D exchange kinetics in the fully oxidized state, a [C*c*O^Ox^_D_] sample was diluted in H_2_O medium (by a 1∶9 ratio), incubated for time **t** to allow H/D exchange, and then reduced for the spectroscopic measurement. The sample is denoted as [[C*c*O^Ox^_D_]_H,t_]^Rd^. It is noteworthy that the final reduction process is essential as the H/D sensitive vibrational modes are only detectable for the fully reduced enzyme, not the oxidized enzyme [29]. To visualize the progression of the H/D exchange, the [[C*c*O^Ox^_D_]_H,t_]^Rd^ – [[C*c*O^Ox^_D_]_H,0_]^Rd^ difference spectra were calculated, in which [[C*c*O^Ox^_D_]_H,0_]^Rd^ was obtained at t = 0 min (i.e. the reduction was carried out immediately after H_2_O exposure). The data obtained at t = 4, 8 and 16 min are shown in Figure 3, in which the [C*c*O^Ox^_H_]^Rd^ – [C*c*O^Ox^_D_]^Rd^ spectrum is also displayed as a reference. In the protocol used in these measurements, if the H/D exchange reaches completion at t = 0, a featureless flat line is expected in the difference spectra. On the other hand, if the H/D exchange takes place in the monitored time window, the difference spectrum will grow in intensity, approaching the level of the reference spectrum (See File S1 for details).

The data show that, in the [[C*c*O^Ox^_D_]_H,t_]^Rd^–[[C*c*O^Ox^_D_]_H,0_]^Rd^ difference spectra, the amplitude of all the heme *a* peaks progressively increased as a function of time, indicating an increasing level of H/D exchange. The spectral shape of the heme *a* modes remained the same as a function of time, indicating that the H/D exchange near the two heme *a* propionates (heme *a*-Pr_A_ and heme *a*-Pr_D_) occurred at the same rate. The time dependence of the degree of H/D exchange calculated from the spectral change is plotted in the top panel of Figure 3. The rate constant (*k*) of the H/D exchange was determined to be 0.23±0.03 min^−1^ (at 23±1°C). In contrast, all of the heme *a*_3_ peaks observed in the reference spectrum are absent (except the small residual *a*_3_-Pr_A_ mode at 1179 cm^−1^) within the 0–16 min time window. The data indicate that H/D exchange near the heme *a*_3_ propionates reached completion at t = 0 min. However, it is important to note that it is unclear if the fast H/D exchange near the heme *a*_3_ propionates occurred exclusively in the oxidized state, considering the fact that the spectroscopic measurement was made in the reduced state in which the H/D exchange near heme *a*_3_ occurs rapidly (Figure 2). Nonetheless, the data confirm that there is no H/D exchange crosstalk between the two heme sites.

An equivalent set of experiments was carried out by diluting the protonated enzyme in the oxidized form, [C*c*O^Ox^_H_] into the D_2_O buffer. Similar spectral changes were observed (Figure S5), although the rate of the H/D exchange near the heme *a* propionates was slightly slower (*k* = 0.10±0.02 min^−1^, see lower trace in top panel of Figure 3), plausibly due to an H/D kinetic isotope effect. Taken together, the data demonstrate that H/D exchange near the heme *a* propionates is prohibited in the reduced state, but can occur with a rate constant of 0.23±0.03 min^−1^ in the oxidized state, while the H/D exchange near the heme *a*_3_ propionates instantaneously reaches near completion in the reduced state.

It has been established that, immediately following turnover of C*c*O, the enzyme is converted to a fully oxidized form, called the “pulsed form”, that is more active as compared to the resting form of the enzyme [37]. To evaluate if the H/D exchange occurring in the pulsed enzyme differs from that in the resting enzyme, similar experiments as described above were carried out with the pulsed enzyme. It was found that the H/D exchange in the pulsed enzyme is the same as that observed in the resting enzyme (File S1 and Figure S6), indicating that there is no difference in the proton accessibility to the heme *a* and heme *a_3_* sites in the two equilibrium forms of the fully oxidized enzyme.

### Effect of the Oxidation and Coordination States of Heme *a*_3_ on the H/D Exchange Near the Heme *a* Site

To determine if the oxidation and coordination states of the heme *a*_3_ affect the H/D exchange rate near the heme *a* site, several bC*c*O samples were prepared and compared (Table S1). To evaluate the effect of the oxidation and coordination states of heme *a*_3_ on the *reduced* heme *a*, [*a* ^2+^+***a*** **_3_^2+^-CO**] and [*a* ^2+^+***a*** **_3_^3+^-SH^−^**] (the fully reduced CO derivative (Figure S2) and the mixed valence SH^−^ derivative (Figure S7), respectively) were examined. We found that the H/D exchange at heme *a* is blocked in both cases, independent of the oxidation and coordination states of heme *a*_3_. To evaluate the effect of oxidation and coordination state of heme *a*_3_ on the *oxidized* heme *a*, the **P_M_** species [2], [*a* ^3+^+***a*** **_3_^4+^ = O^2−^**], was compared to the fully oxidized enzyme, [*a* ^3+^+***a*** **_3_^3+^**] (Figure S8). We found that in the **P_M_** species the H/D exchange near the heme *a* site took place with a rate constant of 0.3±0.1 min^−1^, similar to that observed in the fully oxidized enzyme (*k* = 0.23±0.03 min^−1^). Overall, the data demonstrate that the H/D exchange near heme *a* in both the *reduced* and the *oxidized* state is independent of the oxidation and coordination state of the heme *a*_3_.

### H/D Exchange during Enzymatic Turnover

To determine if the H/D exchange near the heme centers during enzymatic turnover is distinct from that identified under the equilibrium conditions, the deuterated enzyme, reduced with ascorbate (50 mM) and cyt *c* (10 µM), [C*c*O^Ox^_D_]^Rd^, was immersed in the H_2_O buffer and exposed to air for ∼60 s to allow for the turnover of the enzyme (in H_2_O). The resulting oxidized sample, [[[C*c*O^Ox^_D_]^Rd^]_H_]^Ox^, was then purged for 60 s so as to be reduced by the residual ascorbic acid, [[[[C*c*O^Ox^_D_]^Rd^]_H_]^Ox^]^Rd^, for the spectral measurements. To visualize the H/D exchange during the turnover, the [C*c*O^Ox^_H_]^Rd^–[[[[C*c*O^Ox^_D_]^Rd^]_H_]^Ox^]^Rd^ difference spectrum was calculated and is displayed in Figure 2 (Spectrum d). The difference spectrum shows that there is no exchange near heme *a*, under these turnover conditions. However, H/D exchange near the propionates of heme *a*_3_ is complete, presumably due to exchange in the initial reduced enzyme, based on the equilibrium measurements described above. The same results were obtained with longer exposure to air (∼120 s) or when the re-reduction cycle was repeated twice.

These turnover results are consistent with the equilibrium H/D exchange experiments in which rapid exchange occurs near heme *a*_3_ under all conditions and exchange near heme *a* only occurs in the oxidized state with longer exposure times than that which was employed in this experiment (∼60–120 s). Similar results were obtained when dithionite was used as the reductant rather than ascorbate/cyt *c*.

## Discussion

The H/D exchange resonance Raman spectroscopic data reported here demonstrate that (*i*) in the equilibrium reduced state, the H/D exchange near heme *a_3_* occurs rapidly, while that near heme *a* is inhibited, (*ii*) the H/D exchange near heme *a* is enabled when heme *a* is oxidized, (*iii*) the H/D exchange in the heme *a* site is independent of the oxidation and ligation state of heme *a*_3_, (*iv*) there is no H/D exchange crosstalk between the two heme sites.

The process associated with H/D exchange observed in the equilibrium states of bC*c*O described here is fundamentally different from that associated with the proton translocation coupled to the oxygen chemistry carried out by the enzyme. Proton translocation is an active process driven by the free energy released from the O_2_ reduction chemistry [2], which involves a unidirectional proton movement through the protein matrix, against a proton concentration gradient and membrane potential. On the other hand, the H/D exchange observed in the equilibrium states is a passive entropy-driven process, which involves spontaneous migration of protons/deuterons through H-bonding networks linking amino acids and/or water molecules. However, these equilibrium measurements can reveal potential gates and loading sites as well as allowed and restricted proton pathways in the enzyme, all of which play functional roles in proton translocation.

### Proton Accessibility to the Heme *a* and Heme *a*_3_ Sites

Structural and computational studies of bC*c*O and other CcOs show that, in both the reduced and oxidized states, the heme *a*_3_ propionates are connected to the **p**-side surface [16], [38], [39] via an extended H-bonding network involving a large cluster of water molecules (Figure S9). It suggests that the fast H/D exchange observed at the heme *a*_3_ site is a result of proton/deuterium entry through the H-bonding network near the **p**-side surface. On the other hand, it has been proposed that proton access to the heme *a* propionates is blocked from the **p**-side surface due to the keto conformation of the Y440-S441 peptide bond but proton movement from the propionate region to the p-side surface can occur due to the keto-enol tautomerism [18]. This unidirectional pathway was supported by density-functional calculations [40]. Based on those results, the observed redox-controlled H/D exchange near the heme *a* site has to be mediated by proton/deuterium entry from the **n**-side surface likely via the H-channel, the only channel that passes through the heme *a* region (Figure 1) [2],[19]. Previous mutagenesis [18] and structural [19] studies showed that the H-channel (Figure 1) starts from H413 near the **n**-side surface of the enzyme, extends through a series of water accessible cavities (lying alongside the heme *a* farnesyl sidechain), S382 and the OH group of the farnesyl sidechain [17], into the region near the heme *a* formyl and R38 moieties (which are linked together by a H-bond), via a series of amino acids with labile protons [18], [19], and ends at D51 near the **p**-side surface.

**Figure 4:**
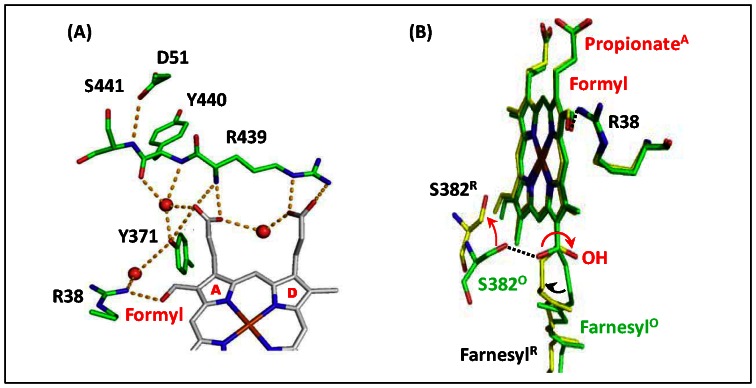
Structure of Heme *a* site in bC*c*O. (A) shows the H-bonding network between the propionate groups of heme *a* and the amino acids/water surrounding it in fully oxidized bC*c*O (PDB: 3ABL). The red spheres indicate water molecules. (B) shows the comparison of heme *a* in the fully oxidized enzyme (PDB: 3ABL) and fully reduced enzyme (PDB: 2EIJ), which are highlighted in green and yellow, respectively. The dotted lines indicate the H-bonds present in the fully oxidized state; the red arrows indicate the structural changes induced by heme reduction. The structural alignment was made by superimposing the heme iron atoms. The structures were drawn with the PyMol Molecular Graphics System, Schrodinger, LLC.

The propionate groups of both hemes interact with their surroundings via strong H-bonding networks [19]. The Pr_D_ group of heme *a* forms H-bonds with the sidechain groups of R439–R438 (Figure 4A; R438 was not shown for clarity) and it is indirectly linked to the Pr_A_ group via H-bonding interactions mediated by a water molecule. Additionally, the Pr_A_ group of heme *a* interacts directly with the backbone moiety of R439–Y440 and the sidechain group of Y371 via an extended H-bonding network and indirectly with the sidechain group of R38 via an intervening water molecule. The sidechain groups of R439–R438 also form H-bonds with both Pr_A_ and Pr_D_ groups of heme *a*_3_. Electrostatic calculations showed that both propionates of heme *a*_3_ and the Pr_D_ group of heme *a* are deprotonated under neutral conditions in both oxidation states [41]; as such they are able to establish strong salt bridges with R438–R439. The observation that the H/D exchange taking place in the heme *a* site is independent of that in the heme *a*_3_ site may be attributed to a robust barrier created by the strong salt bridges between the hemes and R438–R439 that prevents proton leakage between the heme *a* and heme *a*_3_ sites.

Crystallographic studies of bC*c*O [19] showed that the reduction of the enzyme from the oxidized to reduced state introduces a significant conformational change to the farnesyl sidechain of heme *a* (Figure 4B), including ∼180^o^ rotation of its OH group and the movement of S382 away from it, thereby disrupting the H-bond linking the two groups (as reflected by the increase in their distance from 2.8 to 7.6 Å). Resonance Raman data [25] revealed that the reduction of the enzyme also leads to significant strengthening of the H-bond between heme *a* formyl and R38 (which corresponds to an increase in the bond energy by ∼2–2.5 kcal/mol based on the Badger-Bauer rule), although the difference is not evident in the crystallographic data, due to insufficient distance sensitivity [17]. It is noteworthy that the importance of R38 in proton translocation is supported by the observation that the mutation of R54 in *P. denitrificans* C*c*O (equivalent to R38 in bC*c*O) to methionine inhibits its proton pumping activity [24]. The observation that the H/D exchange near heme *a* is inhibited in the reduced state, but becomes allowed in the oxidized state (Figures 2 & 3), suggests that proton accessibility to the heme *a* site is gated by the redox-linked conformational change: the gate only opens when the H-bond between heme *a* formyl and R38 is loosened and the H-bond between farnesyl OH and S382 is intact. (Hereafter, the conformations of the heme *a* farnesyl sidechain and formyl group associated with the oxidized and reduced enzyme are referred as the “open” and “closed” conformation, respectively.).

The observation that protons have gated access to the region near the heme *a* propionate groups suggests that this region constitutes a critical proton loading site that stores protons delivered from the **n**-side surface of the enzyme before they are released into the **p**-side surface and that proton movement in-to and out-of the proton loading site is controlled by the redox state of heme *a*. At present, our data cannot distinguish whether the loading site is associated with the Pr_A_ or the Pr_D_ moieties of heme *a*, as no differences between the behavior of the two groups were identified (plausibly due to their close coupling mediated by the water molecule that links them together shown in Figure 4A). However, importantly, electrostatic calculations by Song *et al*. showed that the heme *a*-Pr_D_ group is deprotonated in both oxidation states whereas the heme *a*-Pr_A_ group is partially protonated in the reduced state under physiological conditions, but deprotonated in the oxidized state, [41]. Likewise, hybrid density functional calculations by Siegbahn *et al*. showed that the heme *a*-Pr_A_ group is protonated in the reduced state [42]. On the basis of our data and the theoretical calculations, in the model described below, we postulate that the loading site is associated with the Pr_A_ group of heme *a*.

### A Postulated Molecular Model for Proton Translocation

**Figure 5:**
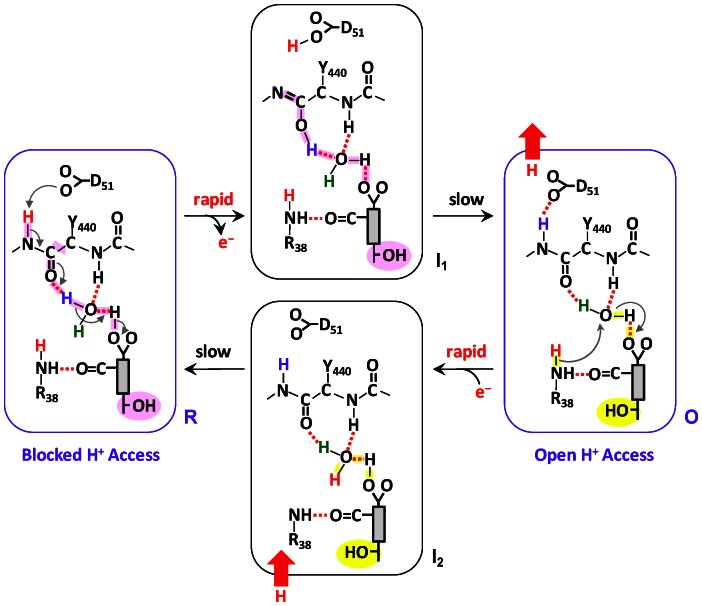
Mechanistic model for proton translocation in bC*c*O. The gray rectangular box indicates the heme *a*, with the farnesyl sidechain, formyl and propionate A groups attached to it. The OH group of the farnesyl sidechain in the “open” and “closed” conformations (see text) is highlighted in yellow and pink, respectively.

Based on the new findings reported here, we propose a molecular model for redox-controlled proton translocation in bC*c*O as illustrated in Figure 5. In the fully reduced enzyme, **R**, we postulate that the heme *a* Pr_A_ is protonated, as suggested by the theoretical calculations [41], [42] and discussed above, while D51 is deprotonated based on crystallographic structure and FTIR data [43]. Proton accessibility to the region near the heme *a* propionates is blocked from the **n**-side surface, due to the closed conformation of the heme *a* farnesyl sidechain and formyl group; it is also blocked from the **p**-side, owing to the keto conformation of the Y440–S441 peptide bond. Accordingly, there is no H/D exchange in the heme *a* site in the equilibrium **R** state (Figure 2).

When heme *a* becomes oxidized, the change in the charge of the heme iron from +2 to +3 triggers the deprotonation of the heme *a*-Pr_A_ group (the postulated proton loading site) to maintain charge neutrality. It leads to a series of proton transfers from the heme *a*-Pr_A_ group to the carbonyl group of Y440, which promotes the keto → enol transition of the Y440–S441 peptide bond and the transfer of a proton to D51. The proton movement results in an unstable transient state, designated as **I_1_**, which is associated with a frustrated protein structure that is relaxed to the equilibrium **O** state on a much longer time scale. The conformational wave initiated by the redox change in heme *a* [24] associated with the relaxation process induces the change of the heme *a* farnesyl sidechain and formyl group from the closed to the open conformation and resets the enol conformation of the Y440–S441 peptide bond back to the more stable keto conformation. The enol→keto transition triggers the rearrangement of the associated H-bonding network, which allows the ejection of a proton to the **p**-side surface, leaving D51 in a deprotonated state. The new conformation enables proton/water accessibility to the heme *a* site from the **n**-side surface via the H-channel, accounting for the observed H/D exchange in the heme *a* site in the equilibrium **O** state (Figure 3).

When the oxidized heme *a* becomes reduced, the change in the heme iron from +3 to +2 induces rapid protonation of the loading site, the Pr_A_ group, to maintain charge neutrality, by taking up a proton from the **n**-side surface as the farnesyl group is in the open conformation. It leads to the transient **I_2_** state that is subsequently relaxed to the equilibrium **R** state, in which the farnesyl group reverts back to the closed conformation.

In is important to note that, in the model, the rapid proton rearrangement triggered by the redox state changes in heme *a* precedes the slower conformational change associated with the heme *a* farnesyl sidechain. In addition, as suggested by electrostatic calculations [41], it is assumed that the heme *a*-Pr_A_ is deprotonated when the heme *a* is oxidized, but is protonated when the heme *a* is reduced. It is also noteworthy that although D51 is deprotonated in both the **R** and **O** states in the model, the latter assumption is not essential as the proton associated with D51 can be released to the **p**-side surface at any stage of the reaction cycle, provided that the Y440–S441 peptide bond is in the keto conformation. Nonetheless, the function of D51 might be carried out by an amino acid that carries a labile proton other than an aspartate residue, accounting for the fact that D51 is not conserved in bacterial C*c*O enzymes.

Based on the model illustrated in Figure 5, the oxidized to reduced transition of heme *a* triggers the uptake of a proton from the **n**-side surface via the H-channel to the proton loading site constituted by the heme *a*-P_A_ group, while the reduced to oxidized transition of heme *a* induces the release of a proton from the proton loading site to the **p**-side surface. The model suggests that the redox-controlled proton movement is tightly gated by the conformational change in the heme *a* farnesyl sidechain and formyl group and rectified by the Y440–S441 keto-enol diode. We hypothesize that the same redox-controlled proton movement takes place during enzymatic turnover, in particular during the **P**→**F**, **F**→**O**, **O**→**E** or **E**→**R** transition [44], [45], considering the fact that during each transition an electron is delivered from cyt *c* to the binuclear center via heme *a* (i.e. each transition is associated with a reduction-oxidation cycle of heme *a*). On the basis of this mechanism, each time an electron passes through heme *a*, a proton is translocated from the **n**-side surface to the **p**-side surface; in addition, the proton translocation is controlled by the redox state of heme *a* and is independent of the oxidation and coordination state of the heme *a*_3_. This scenario is supported by our experimental observations and is in good agreement with the data reported by others demonstrating that two protons are translocated during the oxidative phase (**P**→**F**→**O**) and two more during the reductive phase (**O**→**E**→**R**) of the C*c*O reaction cycle [45]. The observation that there is no proton leakage between the heme *a* and *a*_3_ sites demonstrates that the two sites are fully insulated from each other to ensure tight coupling between vectorial proton translocation near heme *a* and the oxygen chemistry occurring at the heme *a_3_* site. The model described here is consistent with the heme *a* redox-linked proton translocation model, as originally proposed by Artzatbanov *et al*. [20], and the H-channel proton translocation proposed by Yoshikawa and coworkers [17]. However, in our mechanism the gate at heme *a* from the n-side of the membrane is open in the oxidized state and closed in the reduced state, in contrast to the Yoshikawa model in which the channel is open in the reduced state and closed in the oxidized state based on the opening and closing of water channels [17]. (See File S1 for a more complete comparison of the mechanisms.).

### Conclusion

The present study provides experimental evidence supporting a comprehensive model for proton translocation during catalytic turnover of C*c*O (Figure 5). In the postulated model, during enzymatic turnover, each time heme *a* undergoes an oxidation-reduction cycle a proton is loaded from the **n**-side surface to a critical proton loading site constituted by heme *a*-Pr_A_ and subsequently unloaded from the proton loading site to the **p**-side surface. The proton translocation is controlled by the redox state of heme *a*, regardless of the oxidation and coordination state of the heme *a*_3_, and is gated by the conformational state of the heme *a* farnesyl sidechain and formyl group. The model provides a mechanism allowing tight coupling between the vectorial proton translocation controlled by the redox state of heme *a* and the oxygen chemistry occurring in the binuclear center during enzymatic turnover. Not only does it account for the H/D exchange data presented in this work, but it also rationalizes the fragmental, yet important, data reported in literature in the past several decades, including proton accessibility to heme *a* via the **n**-side surface [20], [46], large conformational changes induced by the redox state change in heme *a* [19], a redox Bohr effect associated with heme *a* [20], [22]–[24], a redox-controlled protonation state change of heme *a*-Pr_A_ [41], unidirectional proton movement regulated by the keto-enol tautomerization of the Y440–S441 peptide bond [40] and the translocation of four protons during the full catalytic cycle [45]. In general, this integrated model supports the concept that proton translocation in bC*c*O likely passes through the H-channel and is tightly controlled by the redox change in heme *a*. Equivalent measurements on bacterial oxidases are underway to determine if the proton translocation mechanism reported here is conserved in the bacterial enzymes.

## Supporting Information

Figure S1 **H_2_O/D_2_O sensitive propionate Raman bands of heme** ***a*** **^2+^ (** ***a*** **^Rd^) and heme** ***a*** **_3_^2+^ (** ***a*** **_3_^Rd^).** Resonance Raman spectra of the fully-reduced form of bC*c*O in H_2_O (blue) and D_2_O (red) were obtained with 441.6 nm excitation. The difference spectrum (H_2_O – D_2_O) (black) is shown with a scale expanded by 3-fold as compared to the parent spectra.
(TIF)

Figure S2 **H_2_O - D_2_O resonance Raman difference spectra of the fully reduced (a) and the reduced-CO (b) forms of bC** ***c*** **O upon 441.6 nm excitation.** The heme *a*_3_ modes are not evident in the difference spectra of the CO-adduct with its Soret transition at ∼430 nm, as with the 441.6 nm excitation wavelength they are not enhanced and only the heme *a* modes are present in the spectrum. The Raman difference spectrum of the protonated reduced-CO sample minus the deuterated reduced-CO exposed to protonated buffer for 180 minutes is shown in (c) illustrates that there is no exchange at heme *a* in reduced-CO derivative. The residual intensity in the line at 1179 cm^−1^ compared to those at 1232 and 1340 cm^−1^ shows incomplete cancellation due to contributions originating from modes in addition to those from *a*_3_^Rd^.
(TIF)

Figure S3 **H/D exchange Resonance Raman difference spectra of bC** ***c*** **O upon diluting the protonated sample into deuterated medium.** Trace (a) shows the reference resonance Raman difference spectra of reduced bC*c*O in protonated buffer minus that in deuterated buffer, [C*c*O^Ox^_H_]^Rd^ – [C*c*O^Ox^_D_]^Rd^. Traces (b) and (c) are the resonance Raman difference spectra of the effect of H/D exchange on fully reduced bC*c*O exposed to deuterated buffer for time, **t**, of 5 and 180 min. The differences are those with respect to the standard deuterated spectrum, [[C*c*O^Ox^_H_]^Rd^]_D_,**_t_** - [C*c*O^Ox^_D_]^Rd^. Trace (d) is a difference spectrum [[C*c*O^Ox^_H_]_D_,_0 min_]^Rd^ - [C*c*O^Ox^_D_]^Rd^, in which the former term denotes a fully reduced sample that was prepared by diluting the protonated oxidized bC*c*O into the D_2_O medium and reduced immediately. The [[[C*c*O^Ox^_H_]_D_,_0 min_]^Rd^ spectrum was also used to calculate the resonance Raman difference spectra shown in Figure S5 as a basis spectrum. Spectrum (d) demonstrates that there is very little H/D exchange near heme *a* in the oxidized to reduced transition.
(TIF)

Figure S4 **H/D exchange Resonance Raman difference spectra of the bC** ***c*** **O samples reduced by ascorbic acid and cytochrome** ***c*** **.** Trace (a) shows the reference resonance Raman difference spectra of reduced bC*c*O in protonated buffer minus that in deuterated buffer, [C*c*O^Ox^_H_]^Rd^ – [C*c*O^Ox^_D_]^Rd^. The samples were reduced by adding 50 mM ascorbic acid and 10 µM cytochrome *c* to anaerobic bC*c*O (∼30 µM) solutions in 100 mM Tris-HCl +0.1% decyl moltoside, pH (pD) 8.5. Traces (b) and (c) are the resonance Raman difference spectra of the effect of H/D exchange on fully reduced bC*c*O exposed to protonated buffer for time, **t**, of 5 and 180 min. The differences are those with respect to the standard protonated spectrum, [C*c*O^Ox^_H_]^Rd^ - [[C*c*O^Ox^_D_]^Rd^]_H_,**_t_**. The initial reduction of the deuterated bC*c*O sample (∼300 µM) was done by adding 50 mM ascorbic acid and 100 µM cytochrome *c*. The protonated buffer used for the dilutions also included 50 mM ascorbic acid to have the same ascorbate concentration as the reference samples.
(TIF)

Figure S5 **The H/D exchange resonance Raman difference spectra of bC** ***c*** **O in fully oxidized bC** ***c*** **O upon diluting the protonated sample into deuterated medium.** The [[C*c*O^Ox^_H_]_D_,_0 min_]^Rd^ – [[C*c*O^Ox^_H_]_D_,**_t_**]^Rd^ difference was obtained from the resonance Raman spectra taken at 4 (blue) 8 (red) and 16 (black) minutes, illustrating the growth of the bands associated with the heme *a* propionates.
(TIF)

Figure S6 **Comparison of the H/D exchange of bC** ***c*** **O in the resting oxidized state and the pulsed oxidized state.** Resonance Raman differences with respect to the [C*c*O^Ox^_D_]^Rd^ spectrum were calculated for the fully reduced bC*c*O samples as a function of the preparation. Trace (a) is the reference difference spectrum. Trace (b) shows the H/D exchange of the resting oxidized state for a 2 minute incubation in deuterated buffer prior to reduction. As a comparison for the difference spectrum shown in trace (b), in trace (c) the pulsed oxidized enzyme was formed and tested for the H/D exchange. This was done by exposing a [C*c*O^Ox^_H_]^Rd^ sample to the air for ∼40 seconds until the sample was fully re-oxidized. The resulted pulsed form in H_2_O medium, [[C*c*O^Ox^_H_]^Rd^]^Ox^, was immediately diluted into the D_2_O medium (by a 1∶9 ratio), allowed to exchange for 2 minutes, and reduced again by sodium dithionite for the resonance Raman measurement. The exchange in the pulsed enzyme is the same as that in the resting enzyme.
(TIF)

Figure S7 **Absence of the H/D exchange at heme** ***a*** **in the mixed valence-SH forms of bC** ***c*** **O.** The [C*c*O^Ox^_H_]^SH̄^ – [C*c*O^Ox^_D_]^SH̄^ reference (c) is compared to [C*c*O^Ox^_H_]^SH̄^ – [[C*c*O^Ox^_D_]^SH̄^]_H_,**_t_** obtained at 5 (d), 15 (e) and 25 (f) minutes. The data accumulation time of each original spectrum was 10 minutes.
(TIF)

Figure S8 **Progress of the H/D exchange at the heme** ***a*** **in the P_M_ form of bC** ***c*** **O. In the mixed valence P_M_ form, the heme** ***a*** **_3_ is a ferryl species (Fe^4+^ = O^2−^) and heme** ***a*** **is in its ferric form.** After the indicated incubations in the **P_M_** forms, the samples were reduced in the presence of the residual CO. The [[C*c*O**^PM^**_D_]_H_,**_t_**]^Rd/CO^ – [[C*c*O**^PM^**_D_]_H_,_0 min_]^Rd/CO^ difference was obtained for the resonance Raman spectra taken at 4 (blue) and 8 (red) minutes.
(TIF)

Figure S9 **The Hydrogen bonding network in the region of the heme** ***a*** **_3_ propionate groups in oxidized bC** ***c*** **O (PDB: 3ABL).** The water molecules identified in the crystal structure are shown as red spheres. The yellow dotted lines show H-bonding interactions.
(TIF)

Table S1 **H/D Exchange at heme** ***a*** **in bC** ***c*** **O for various derivatives.** The results illustrate that the oxidation and coordination states of heme *a*_3_ do not affect the exchange properties at heme *a*.
(PDF)

File S1 **This file contains the following information:** 1) Definitions of all of the symbols used in the manuscript; 2) Calculation of the changes in intensity in the resonance Raman difference spectra of the reduced enzyme (Figure 2 in the main text); 3) Calculation of the changes in intensity in the resonance Raman difference spectra of the oxidized enzyme (Figure 3 in the main text); 4) Effects of the heme *a*_3_ redox/coordination/spin status on the H/D exchange at heme *a*; 5) H/D exchange in the pulsed enzyme; 6) The role of water channels in proton translocation.
(PDF)
