# Supplementary figures and images for: Redox-Controlled Proton Gating in Bovine Cytochrome c Oxidase
Source: PLoS One. 2013 May 16;8(5):e63669. doi: 10.1371/journal.pone.0063669 (PMC3656056; doi:10.1371/journal.pone.0063669)

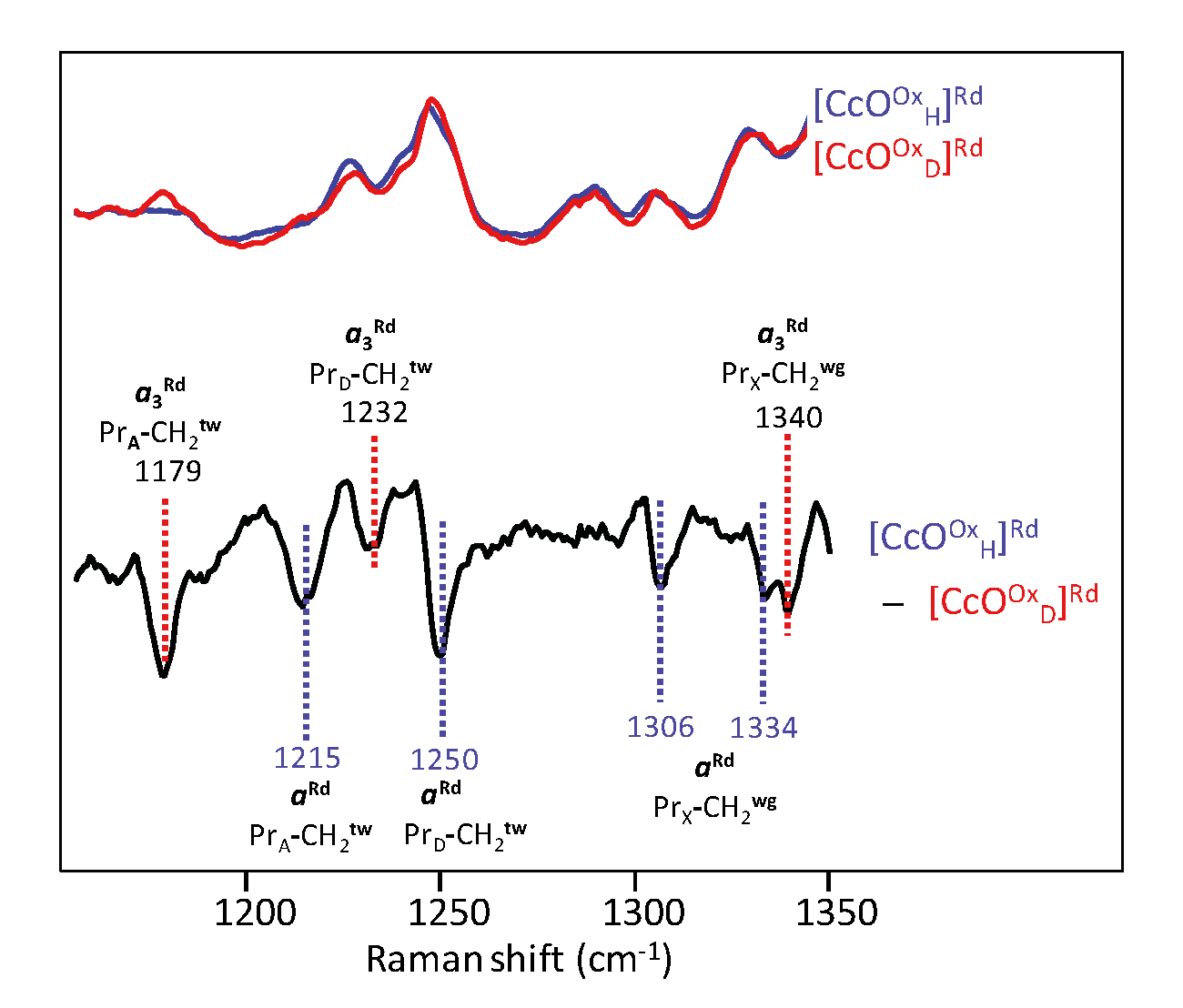

Supplement: Figure S1 — H2O/D2O sensitive propionate Raman bands of heme a 2+ ( a Rd) and heme a 32+ ( a 3Rd). Resonance Raman spectra of the fully-reduced form of bCcO in H2O (blue) and D2O (red) were obtained with 441.6 nm excitation. The difference spectrum (H2O – D2O) (black) is shown with a scale expanded by 3-fold as compared to the parent spectra. (TIF) [file pone.0063669.s001.tif]

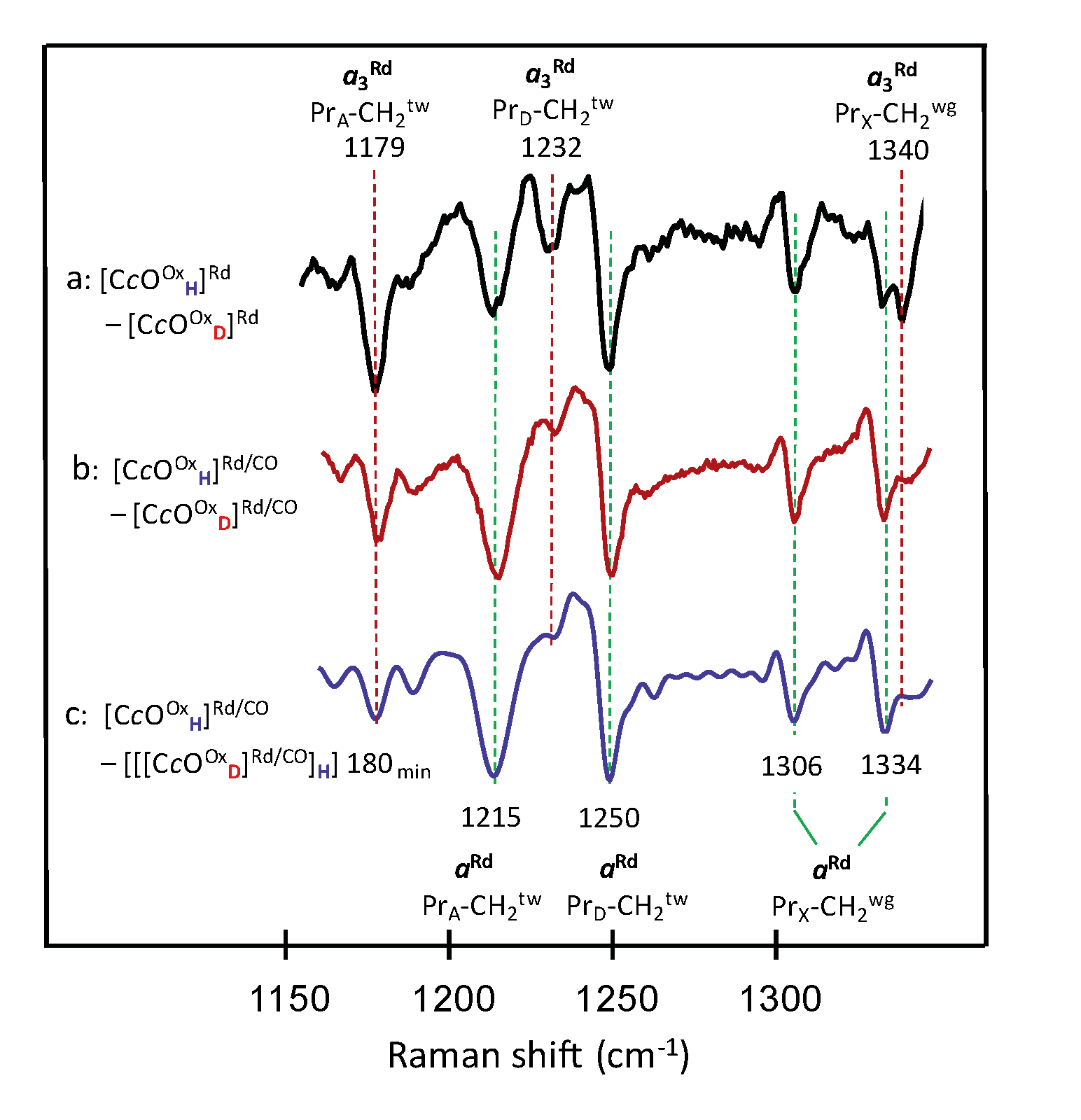

Supplement: Figure S2 — H2O - D2O resonance Raman difference spectra of the fully reduced (a) and the reduced-CO (b) forms of bC c O upon 441.6 nm excitation. The heme a 3 modes are not evident in the difference spectra of the CO-adduct with its Soret transition at ∼430 nm, as with the 441.6 nm excitation wavelength they are not enhanced and only the heme a modes are present in the spectrum. The Raman difference spectrum of the protonated reduced-CO sample minus the deuterated reduced-CO exposed to protonated buffer for 180 minutes is shown in (c) illustrates that there is no exchange at heme a in reduced-CO derivative. The residual intensity in the line at 1179 cm−1 compared to those at 1232 and 1340 cm−1 shows incomplete cancellation due to contributions originating from modes in addition to those from a 3 Rd. (TIF) [file pone.0063669.s002.tif]

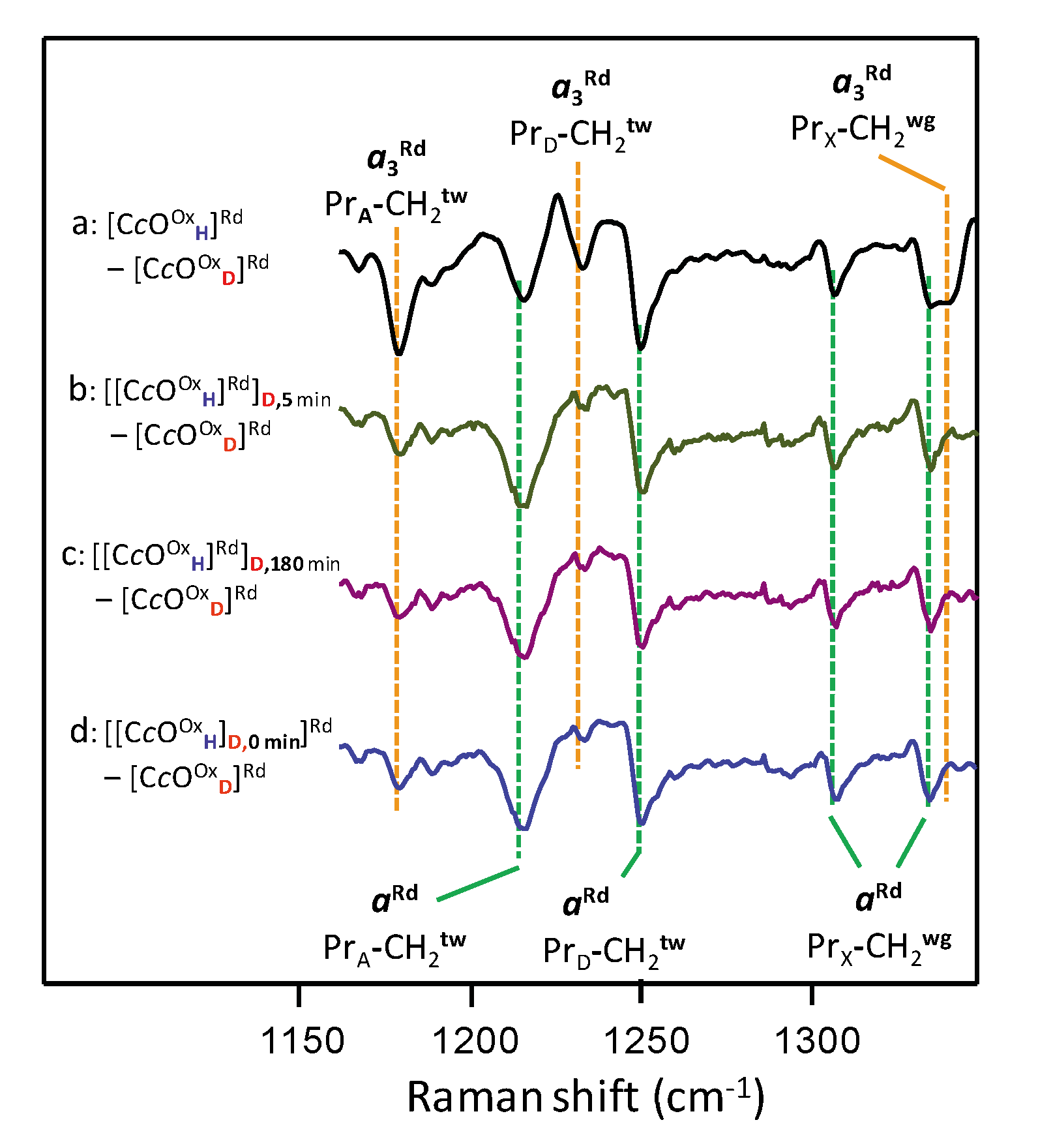

Supplement: Figure S3 — H/D exchange Resonance Raman difference spectra of bC c O upon diluting the protonated sample into deuterated medium. Trace (a) shows the reference resonance Raman difference spectra of reduced bCcO in protonated buffer minus that in deuterated buffer, [CcOOx H]Rd – [CcOOx D]Rd. Traces (b) and (c) are the resonance Raman difference spectra of the effect of H/D exchange on fully reduced bCcO exposed to deuterated buffer for time, t, of 5 and 180 min. The differences are those with respect to the standard deuterated spectrum, [[CcOOx H]Rd]D,t - [CcOOx D]Rd. Trace (d) is a difference spectrum [[CcOOx H]D,0 min]Rd - [CcOOx D]Rd, in which the former term denotes a fully reduced sample that was prepared by diluting the protonated oxidized bCcO into the D2O medium and reduced immediately. The [[[CcOOx H]D,0 min]Rd spectrum was also used to calculate the resonance Raman difference spectra shown in Figure S5 as a basis spectrum. Spectrum (d) demonstrates that there is very little H/D exchange near heme a in the oxidized to reduced transition. (TIF) [file pone.0063669.s003.tif]

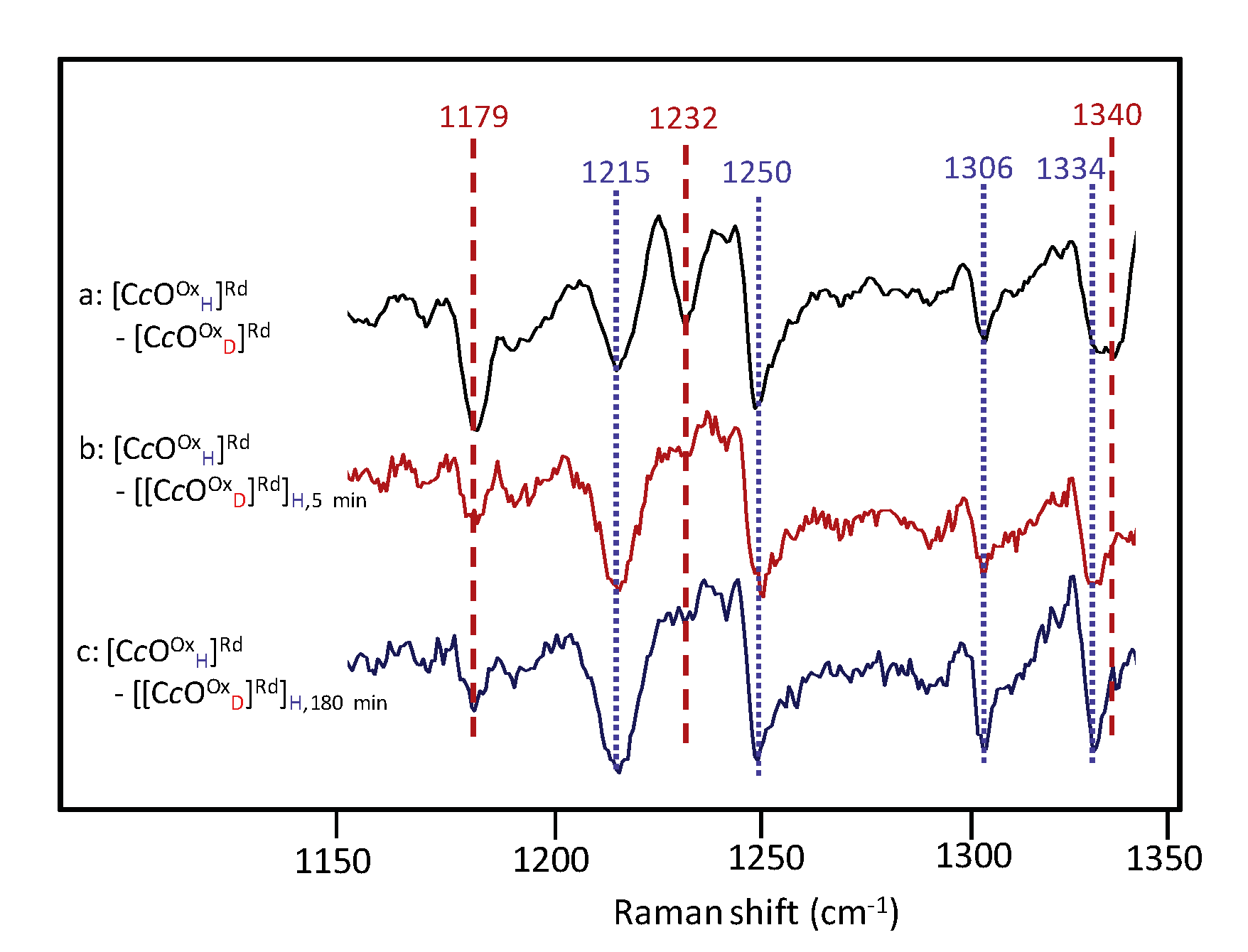

Supplement: Figure S4 — H/D exchange Resonance Raman difference spectra of the bC c O samples reduced by ascorbic acid and cytochrome c . Trace (a) shows the reference resonance Raman difference spectra of reduced bCcO in protonated buffer minus that in deuterated buffer, [CcOOx H]Rd – [CcOOx D]Rd. The samples were reduced by adding 50 mM ascorbic acid and 10 µM cytochrome c to anaerobic bCcO (∼30 µM) solutions in 100 mM Tris-HCl +0.1% decyl moltoside, pH (pD) 8.5. Traces (b) and (c) are the resonance Raman difference spectra of the effect of H/D exchange on fully reduced bCcO exposed to protonated buffer for time, t, of 5 and 180 min. The differences are those with respect to the standard protonated spectrum, [CcOOx H]Rd - [[CcOOx D]Rd]H,t. The initial reduction of the deuterated bCcO sample (∼300 µM) was done by adding 50 mM ascorbic acid and 100 µM cytochrome c. The protonated buffer used for the dilutions also included 50 mM ascorbic acid to have the same ascorbate concentration as the reference samples. (TIF) [file pone.0063669.s004.tif]

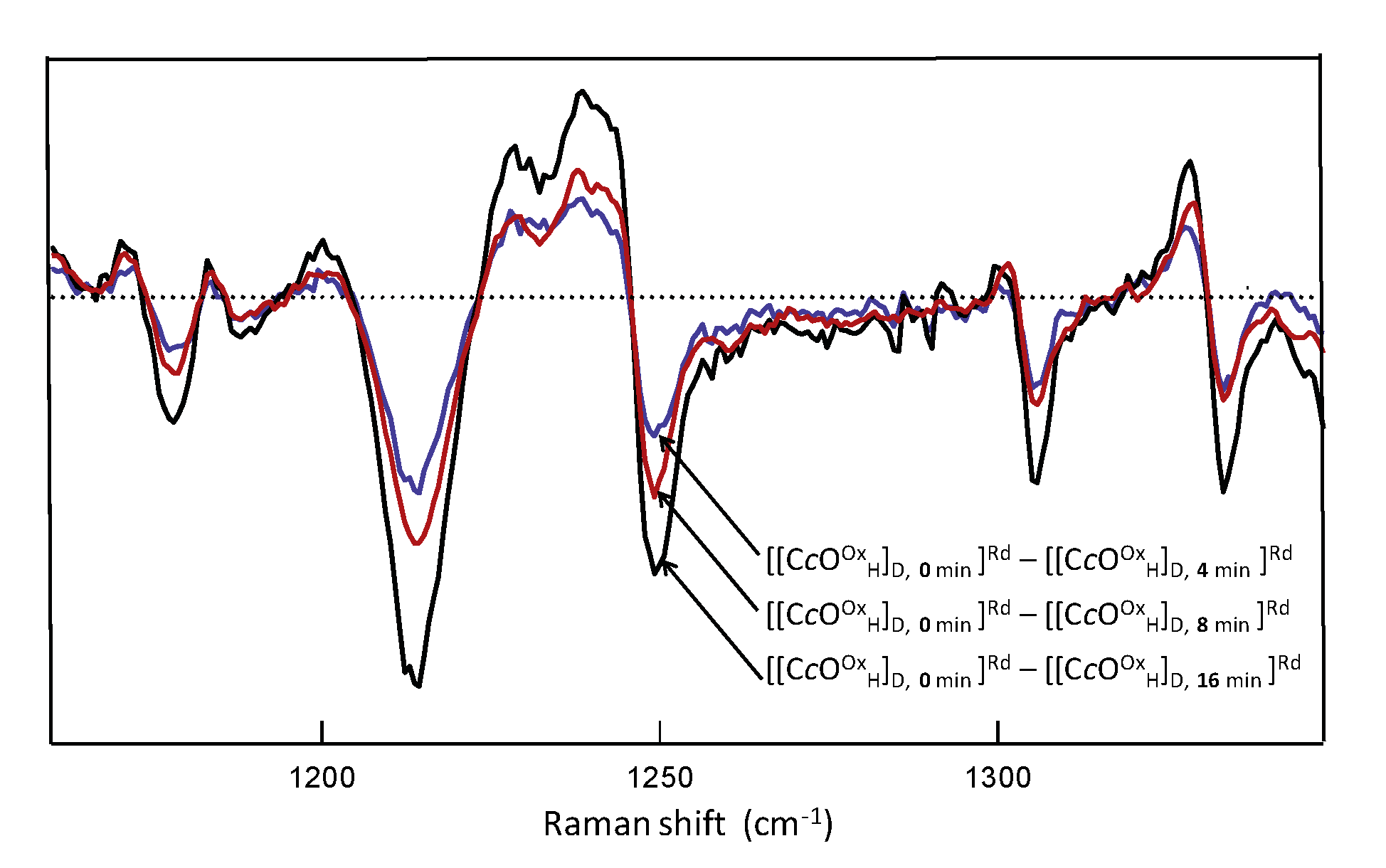

Supplement: Figure S5 — The H/D exchange resonance Raman difference spectra of bC c O in fully oxidized bC c O upon diluting the protonated sample into deuterated medium. The [[CcOOx H]D,0 min]Rd – [[CcOOx H]D,t]Rd difference was obtained from the resonance Raman spectra taken at 4 (blue) 8 (red) and 16 (black) minutes, illustrating the growth of the bands associated with the heme a propionates. (TIF) [file pone.0063669.s005.tif]

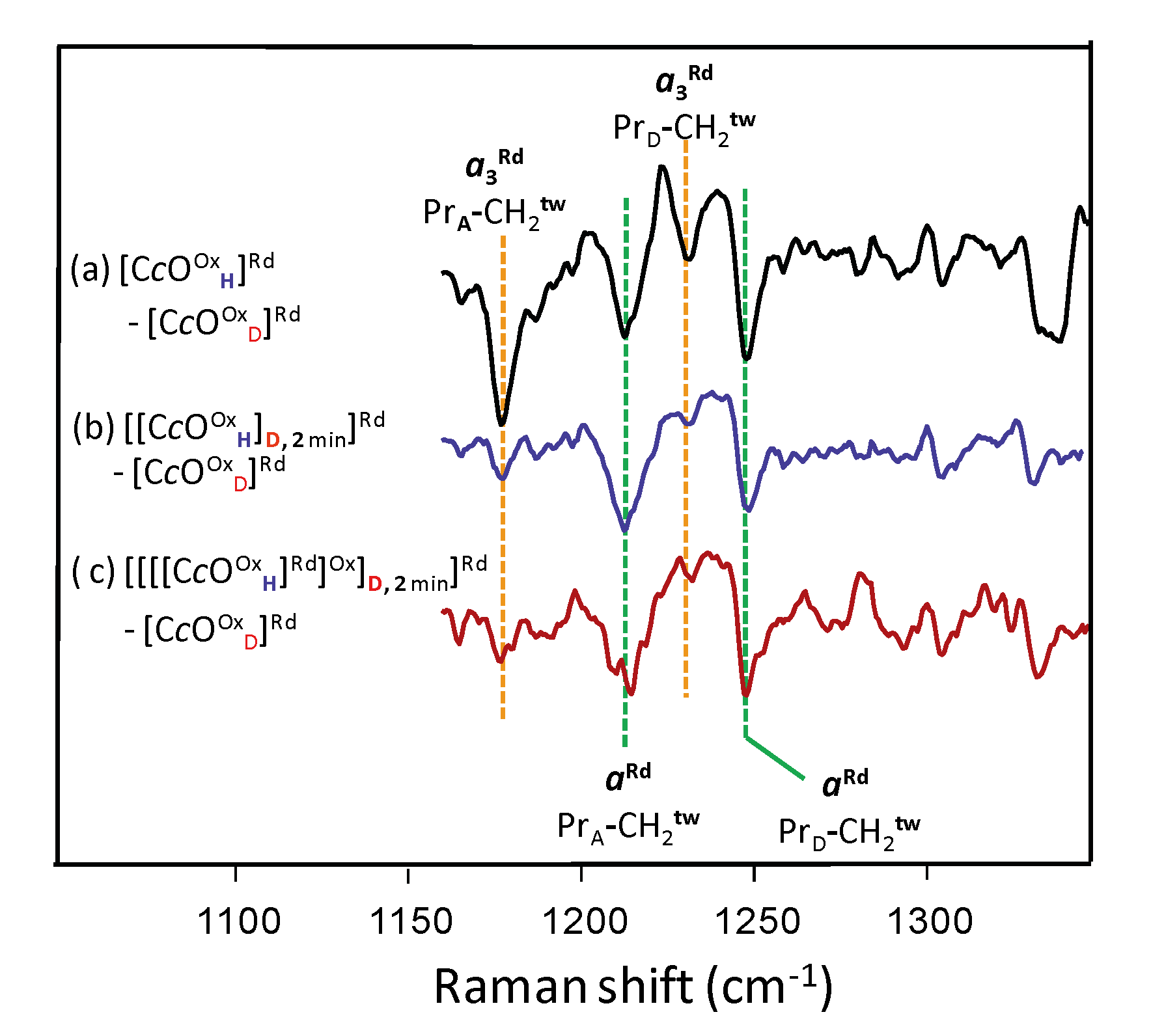

Supplement: Figure S6 — Comparison of the H/D exchange of bC c O in the resting oxidized state and the pulsed oxidized state. Resonance Raman differences with respect to the [CcOOx D]Rd spectrum were calculated for the fully reduced bCcO samples as a function of the preparation. Trace (a) is the reference difference spectrum. Trace (b) shows the H/D exchange of the resting oxidized state for a 2 minute incubation in deuterated buffer prior to reduction. As a comparison for the difference spectrum shown in trace (b), in trace (c) the pulsed oxidized enzyme was formed and tested for the H/D exchange. This was done by exposing a [CcOOx H]Rd sample to the air for ∼40 seconds until the sample was fully re-oxidized. The resulted pulsed form in H2O medium, [[CcOOx H]Rd]Ox, was immediately diluted into the D2O medium (by a 1∶9 ratio), allowed to exchange for 2 minutes, and reduced again by sodium dithionite for the resonance Raman measurement. The exchange in the pulsed enzyme is the same as that in the resting enzyme. (TIF) [file pone.0063669.s006.tif]

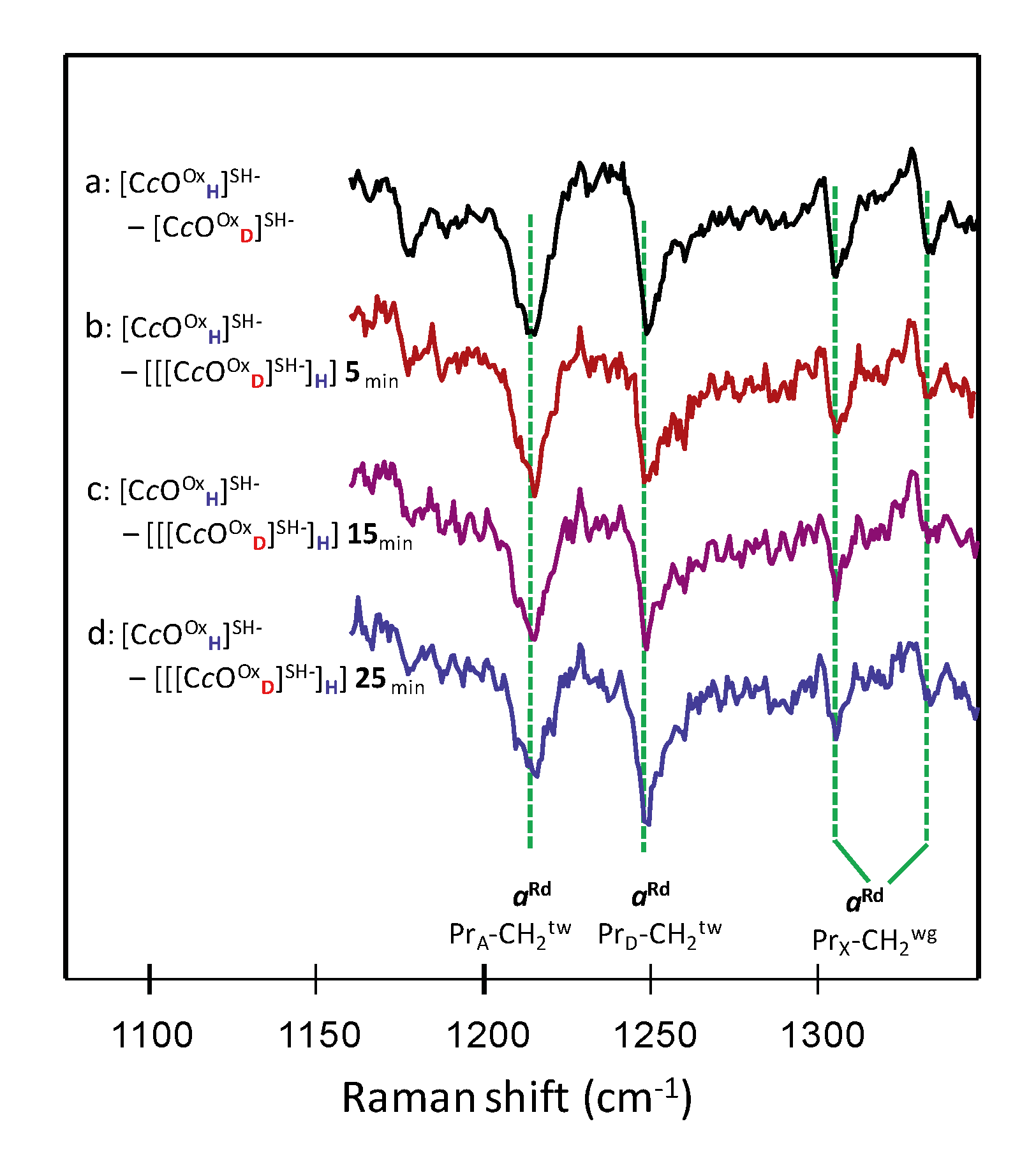

Supplement: Figure S7 — Absence of the H/D exchange at heme a in the mixed valence-SH forms of bC c O. The [CcOOx H]SH̄ – [CcOOx D]SH̄ reference (c) is compared to [CcOOx H]SH̄ – [[CcOOx D]SH̄]H,t obtained at 5 (d), 15 (e) and 25 (f) minutes. The data accumulation time of each original spectrum was 10 minutes. (TIF) [file pone.0063669.s007.tif]

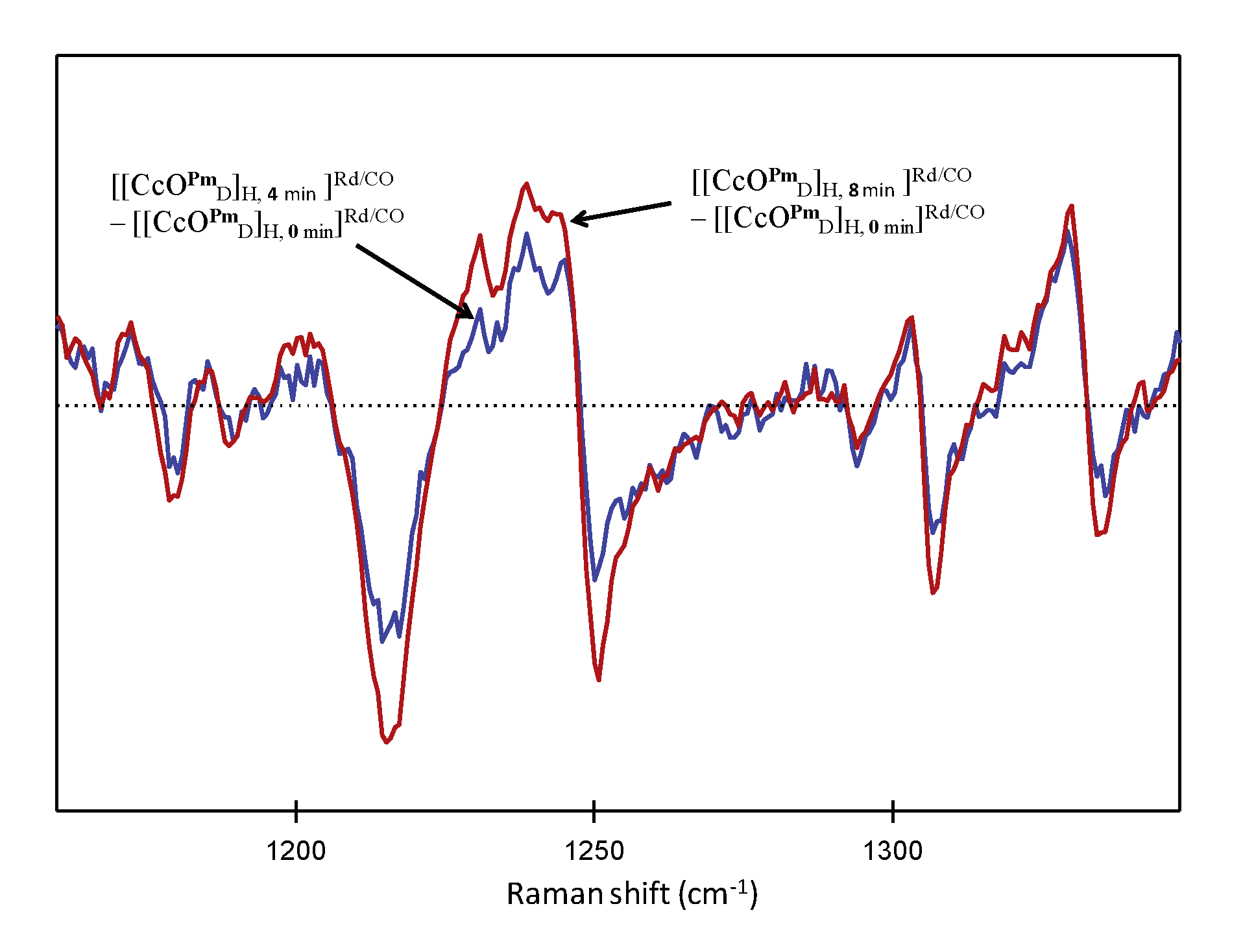

Supplement: Figure S8 — Progress of the H/D exchange at the heme a in the PM form of bC c O. In the mixed valence PM form, the heme a 3 is a ferryl species (Fe4+ = O2−) and heme a is in its ferric form. After the indicated incubations in the PM forms, the samples were reduced in the presence of the residual CO. The [[CcOPM D]H,t]Rd/CO – [[CcOPM D]H,0 min]Rd/CO difference was obtained for the resonance Raman spectra taken at 4 (blue) and 8 (red) minutes. (TIF) [file pone.0063669.s008.tif]

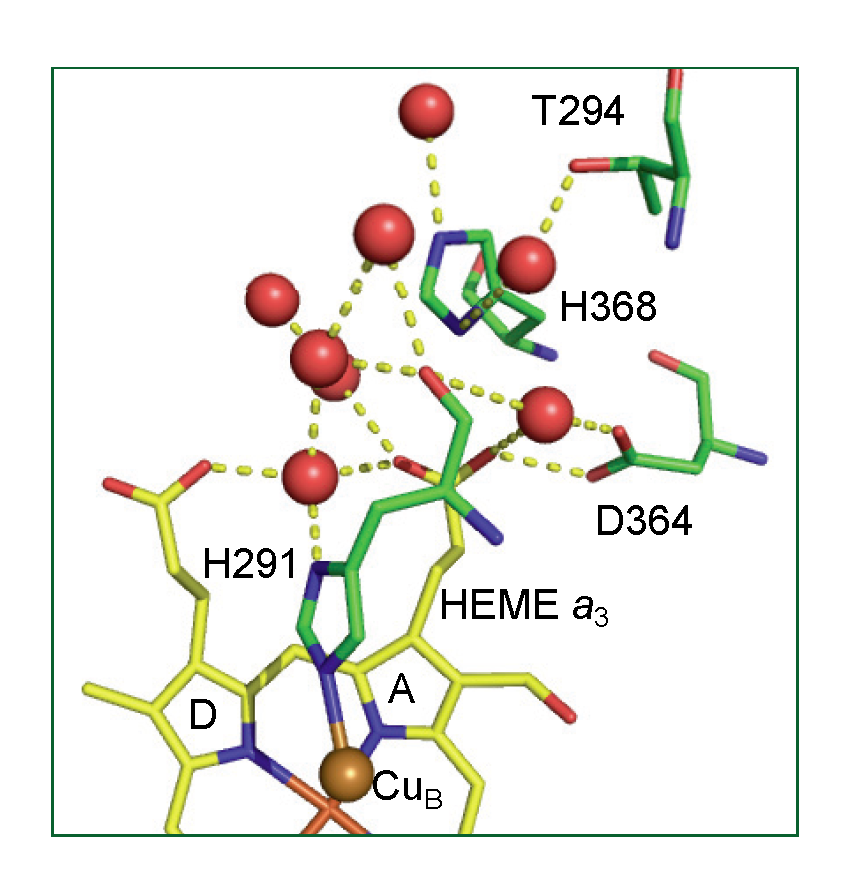

Supplement: Figure S9 — The Hydrogen bonding network in the region of the heme a 3 propionate groups in oxidized bC c O (PDB: 3ABL). The water molecules identified in the crystal structure are shown as red spheres. The yellow dotted lines show H-bonding interactions. (TIF) [file pone.0063669.s009.tif]
