# Supplementary material for: Redox-Controlled Proton Gating in Bovine Cytochrome c Oxidase
Source: PLoS One. 2013 May 16;8(5):e63669. doi: 10.1371/journal.pone.0063669 (PMC3656056; doi:10.1371/journal.pone.0063669)
Supplement: Table S1 — H/D Exchange at heme a in bC c O for various derivatives. The results illustrate that the oxidation and coordination states of heme a 3 do not affect the exchange properties at heme a. (PDF) [file pone.0063669.s010.pdf]

Table S1 H/D Exchange at heme *a* in bC*c*O for various derivatives. The results illustrate that the oxidation and coordination states of heme a_3 do not affect the exchange properties at heme *a*.

Forms	heme *a*	heme a_3	a_3 coordination/spin[a]	*a* H/D exchange[b]
MV-SH^-	a^{2+}	a_3^{3+}- SH^-	6cls	Blocked
Reduced-CO	a^{2+}	a_3^{2+}-CO	6cls	Blocked
Fully-Reduced	a^{2+}	a_3^{2+}	5chs	Blocked
Resting	a^{3+}	a_3^{3+}	5chs	$k = 2.3\ (\pm\ 0.3) \times 10^{-1}\ min^{-1}$
P_M	a^{3+}	a_3^{4+}=O^{2-}	6cls	$k = 3\ (\pm\ 1) \times 10^{-1}\ min^{-1}$

a: The coordination and spin states of the heme a_3 are denoted as: 5chs, five coordinate and high spin; 6cls, six coordinate and low spin. b: The rate constant listed are those for the D to H exchange at 23°C.
