# Supplementary material for: Redox-Controlled Proton Gating in Bovine Cytochrome c Oxidase
Source: PLoS One. 2013 May 16;8(5):e63669. doi: 10.1371/journal.pone.0063669 (PMC3656056; doi:10.1371/journal.pone.0063669)
Supplement: File S1 — This file contains the following information: 1) Definitions of all of the symbols used in the manuscript; 2) Calculation of the changes in intensity in the resonance Raman difference spectra of the reduced enzyme (Figure 2 in the main text); 3) Calculation of the changes in intensity in the resonance Raman difference spectra of the oxidized enzyme (Figure 3 in the main text); 4) Effects of the heme a 3 redox/coordination/spin status on the H/D exchange at heme a; 5) H/D exchange in the pulsed enzyme; 6) The role of water channels in proton translocation. (PDF) [file pone.0063669.s011.pdf]

Text S1 This file contains the following information: 1) Definitions of all of the symbols used in the manuscript; 2) Calculation of the changes in intensity in the resonance Raman difference spectra of the reduced enzyme (Figure 2 in the main text); 3) Calculation of the changes in intensity in the resonance Raman difference spectra of the oxidized enzyme (Figure 3 in the main text); 4) Effects of the heme a_3 redox/coordination/spin status on the H/D exchange at heme *a*; 5) H/D exchange in the pulsed enzyme; 6) The role of water channels in proton translocation.

1) Definitions of the symbols.

A: [C*c*$O^{Ox}{}_{H}$] and [C*c*$O^{Ox}{}_{D}$]: Samples obtained by incubating oxidized (Ox) bCcO in protonated and deuterated buffers, respectively, for at least 8 hours.

B: [C*c*$O^{Ox}{}_{H}]^{Rd}$ and [C*c*$O^{Ox}{}_{D}]^{Rd}$: Reduction (Rd) of the samples defined in A.

C: [[C*c*$O^{Ox}{}_{H}]^{Rd}]_{D,\mathbf{t}}$ and [[C*c*$O^{Ox}{}_{D}]^{Rd}]_{H,\mathbf{t}}$: Dilution of the samples defined in B in deuterated and protonated buffers, respectively, and then incubated for time **t** before the resonance Raman measurements.

D: [[C*c*$O^{Ox}{}_{H}]_{D,\mathbf{t}}]^{Rd}$ and [[C*c*$O^{Ox}{}_{D}]_{H,\mathbf{t}}]^{Rd}$: Dilution of the samples defined in A in deuterated and protonated buffers, respectively, incubated for time **t**, and then reduced before the resonance Raman measurements. In particular, [[C*c*$O^{Ox}{}_{H}]_{D,0min}]^{Rd}$ and [[C*c*$O^{Ox}{}_{D}]_{H,0min}]^{Rd}$ are those reduced immediately after the dilution to determine the exchange during the oxidized to reduced transition.

E: [[[[C*c*$O^{Ox}{}_{H}]^{Rd}]_{D}]^{Ox}]^{Rd}$: A [[[C*c*$O^{Ox}{}_{H}]^{Rd}]_{D}$] sample that was re-oxidized in deuterated buffer to determine the exchange during enzymatic turnover. The resonance Raman measurement was then made after the dithionite reduction of the sample.

F: [C*c*$O^{Ox}{}_{H}]^{Rd/CO}$ and [C*c*$O^{Ox}{}_{D}]^{Rd/CO}$: Samples obtained by reducing [C*c*$O^{Ox}{}_{H}]^{Rd}$ and [C*c*$O^{Ox}{}_{D}]^{Rd}$ in the presence of CO.

G: [C*c*$O^{Ox}{}_{H}]^{SH^{-}}$ and [C*c*$O^{Ox}{}_{D}]^{SH^{-}}$: Samples obtained by partially reducing [C*c*$O^{Ox}{}_{H}$] and [C*c*$O^{Ox}{}_{D}$] by SH^{-}, respectively.

H: $[[CcO^{P_M}{}_{D}]_{H,\mathbf{t}}]^{Rd/CO}$: The $\mathbf{P_M}$ intermediate was formed by exposing the oxidized enzyme to CO/O_2 in deuterated buffer. It was exchanged in protonated buffer for time **t**, and then reduced with the CO still present.

2) *Calculation of the changes in intensity in the resonance Raman difference spectra of the reduced enzyme (Figure 2 in the main text).*

As heme *a* and heme a_3 are not expected to have the same degree of the H/D exchange, their concentrations in the difference spectra may be expressed as follows:

$$[CcO^{Ox}{}_{H}]^{Rd} – [[CcO^{Ox}{}_{D}]^{Rd}]_{H,\mathbf{t}}$$
$$= [a^{2+}{}_{H} + a_3{}^{2+}{}_{H}] - [m\cdot a^{2+}{}_{H} + \{1\text{-}m\}\cdot a^{2+}{}_{D} + n\cdot a_3{}^{2+}{}_{H} + \{1\text{-}n\}\cdot a_3{}^{2+}{}_{D}]$$
$$= \{1\text{-}m\}\cdot[a^{2+}{}_{H} - a^{2+}{}_{D}] + \{1\text{-}n\}\cdot[a_3{}^{2+}{}_{H} - a_3{}^{2+}{}_{D}] \quad \text{Eq. S1}$$

where *m* and *n* are the fractional populations of heme *a* and a_3, respectively, that underwent the D to H exchange within **t**. Therefore, if the exchange is complete, *m* (or *n*) will equal one and the difference spectrum will be featureless. In contrast, if there is no exchange, *m* (or *n*) will be zero and the difference spectrum will be the same as that of the reference difference spectrum.

3) *Calculation of the changes in intensity in the resonance Raman difference spectra of the oxidized enzyme (Figure 3 in the main text).*

Thus, the heme a_3 modes are the same (fully exchanged) in both the reference (at time ~0) and in the time **t** spectrum so they cancel out and the remaining lines reflect the spectral changes in heme *a* only; the difference spectrum equation may then be written as:

$$[[CcO^{Ox}{}_{D}]_{H,\mathbf{t}}]^{Rd} – [[CcO^{Ox}{}_{D}]_{H,0min}]^{Rd}$$
$$= [m'\cdot a^{2+}{}_{H} + \{1\text{-}m'\}\cdot a^{2+}{}_{D} + a_3{}^{2+}{}_{H}] - [a^{2+}{}_{D} + a_3{}^{2+}{}_{H}]$$
$$= m'\cdot[a^{2+}{}_{H} - a^{2+}{}_{D}] \quad \text{Eq. S2}$$

where *m'* represents the relative population of heme *a* that underwent a D to H exchange in the resting state within time, **t**. Based on this formula, the amplitude of the H/D difference peaks of heme a^{2+} increases with increasing time, **t,** as the exchange progresses.

4) *Effects of the heme a_3 redox/coordination/spin status on the H/D exchange at heme a.*

The experiments on the reduced-CO form was done in a manner the same as that employed for the fully-reduced form, except that a $[\mathrm{C}c\mathrm{O}^{\mathrm{Ox}}{}_{\mathrm{D}}]^{\mathrm{Rd/CO}}$ sample instead of the $[\mathrm{C}c\mathrm{O}^{\mathrm{Ox}}{}_{\mathrm{D}}]^{\mathrm{Rd}}$ was diluted into the H_2O medium. The resonance Raman data measured after the dilution was then analyzed by calculating a difference spectrum $[\mathrm{C}c\mathrm{O}^{\mathrm{Ox}}{}_{\mathrm{H}}]^{\mathrm{Rd/CO}}$ – $[[\mathrm{C}c\mathrm{O}^{\mathrm{Ox}}{}_{\mathrm{D}}]^{\mathrm{Rd/CO}}]_{\mathrm{H},\mathbf{t}}$. Analogous to the formula given by eq. 1, the standard H/D difference peaks of the heme a^{2+} will appear in such a difference spectrum, if the H/D exchange at the heme a^{2+} is hindered. In Figure S2, the difference spectrum at 180 minute (b) is compared with the standard H/D difference spectrum (a) for the reduced-CO form. The data show that the H/D exchange at the heme a^{2+} is blocked in the reduced-CO form of the enzyme.

The investigations on the MV-SH^{-} form were somewhat difficult as compared to those on the other forms, because of the stability of the form. The sulfide causes the reduction of heme *a* at one hand, and it binds to the heme a_3^{3+} on the other to form the mixed valence (a^{2+} + a_3^{3+}-SH^{-}) configuration. The successful preparation and the stability of the form depend on the experimental conditions, such as the concentration of the sulfide [1]. In this study, we added 1 mM sodium sulfide aerobically to the $\mathrm{C}c\mathrm{O}^{\mathrm{Ox}}{}_{\mathrm{D}}$ sample, and incubated the sample (for ~15 minutes) until the optical absorption showed the complete formation of the MV-SH^{-} form. The resulted MV-SH^{-} sample was diluted (by 1:9 ratio) into an H_2O buffer, which included 1 mM sodium sulfide. Five minutes after the dilution, resonance Raman spectra were measured repeatedly every 10 minutes. In Figure S7, the resonance Raman data are expressed in a form $[\mathrm{C}c\mathrm{O}^{\mathrm{Ox}}{}_{\mathrm{H}}]^{\mathrm{SH^-}}$ – $[[\mathrm{C}c\mathrm{O}^{\mathrm{Ox}}{}_{\mathrm{D}}]^{\mathrm{SH^-}}]_{\mathrm{H},\mathbf{t}}$. In the difference spectra, the presence of the H/D difference peaks is a measure for the absence of the H/D exchange at the heme a^{2+}. The resonance Raman data showed that the A and D propionate peaks seen in the standard difference spectrum (Figure S7, spectrum (c)) remained after exposure to the protonated buffer from 5-25 minutes. Resonance Raman data taken at time points later than those shown here were not reliable, because the heme *a* in the sample gradually became oxidized (data not shown). Although the time frame of the investigation was limited, the present results show a persistent lack of H/D exchange at heme a^{2+} in the MV-SH^{-} form.

It is well established that the $\mathbf{P_M}$ form of bC*c*O is generated, when resting bC*c*O is exposed to a mixture of CO and O_2 gasses; CO is acting as a reducing agent for heme a_3, while the O_2 molecule is catalyzed at heme a_3^{2+}, leading to the a_3^{4+}=O^{2-} heme [2]. We incubated the CcO^{Ox}_D sample in a 1:1 mixture of CO and O_2 for 15 minutes to make a $\mathbf{P_M}$ preparation (termed $CcO^{\mathbf{P_M}}_D$). The $CcO^{\mathbf{P_M}}_D$ sample was then anaerobically transferred into a Raman cell filled with 100 % CO. In the Raman cell, the sample was diluted anaerobically with the H_2O buffer, incubated for time, **t**, and reduced by dithionite to form $[[CcO^{\mathbf{P_M}}_D]_{H,\mathbf{t}}]^{Rd/CO}$ for the resonance Raman measurements. Since the reduction of the sample was done in the CO atmosphere, the $[[CcO^{\mathbf{P_M}}_D]_{H,\mathbf{t}}]^{Rd/CO}$ preparation was in the reduced-CO form.

We continuously recorded the visible-absorption spectrum for each sample from immediately after the dilution to before the reduction by dithionite, and made sure that the sample was always in the $\mathbf{P_M}$ form during the incubation period. Although the exact extinction coefficients have not reported for the $\mathbf{P_M}$ form made by the present method, its Soret-visible absorption spectrum was given in comparison with other forms of bC*c*O [3]. On the basis of such spectral comparisons, together with known extinction coefficients of bC*c*O, we estimated an extinction coefficient of $\mathbf{P_M}$ form to be 21 mM^{-1} cm^{-1} ($\varepsilon_{604\text{-}630}$), and also determined that of the oxidized form for the same wavelength interval (604 nm to 630 nm) to be 13.6 mM^{-1} cm^{-1}. Using these extinction coefficient values, the population of the $\mathbf{P_M}$ form during the incubation period was determined to be >87 %.

The dilution/reduction sequence described here was the same as that we used for testing the H/D exchange in the resting state, although the starting material before the dilution was $CcO^{\mathbf{P_M}}_D$ instead of CcO^{Ox}_D, while the final product was in the reduced-CO state instead of the fully-reduced state. Accordingly, analogous to the formulation of Eq. S2, we can draw the following scheme for analyzing the resonance Raman spectra from the $[[CcO^{\mathbf{P_M}}_D]_H]\mathbf{t}]^{Rd/CO}$ samples,

$$[[CcO^{\mathbf{P_M}}_D]_{H,\mathbf{t}}]^{Rd/CO} - [[CcO^{\mathbf{P_M}}_D]_{H,0min}]^{Rd/CO}$$
$$= m''\cdot[a^{2+}_H - a^{2+}_D] \qquad \text{Eq. S3,}$$

where *m''* is the population of heme *a* that was subjected to the D to H exchange at the $\mathbf{P_M}$ state within time, **t**. The difference resonance Raman spectra calculated according to the Eq. S3

indeed showed a time dependent increase of the amplitude (Figure S8), which from which the rate of exchange was determined to be 3 (± 1) x 10^{-1} min^{-1}.

5) *H/D exchange in the pulsed enzyme.*

The resting form of C*c*O is converted to a slightly different form, called the "pulsed form", immediately following turnover of the enzyme and that the pulsed form of the oxidized enzyme exhibits a higher activity [4]. To evaluate if the H/D exchange occurring in the pulsed enzyme differs from that in the resting enzyme, two samples were prepared and compared. The sample derived from the pulsed enzyme, denoted as $[[[[CcO^{Ox}{}_{H}]^{Rd}]^{Ox}]_{D,2min}]^{Rd}$, was prepared by reducing the resting enzyme, in H_2O with minimal dithionite and then exposing it to air for ~40 s (to allow turnover of the enzyme) until the enzyme was fully oxidized; the resulting pulsed enzyme was subsequently diluted into the D_2O buffer for 2 minutes prior to its re-reduction to the ferrous state for the spectral measurement. The sample derived from the resting enzyme, denoted as $[[CcO^{Ox}{}_{H}]_{D,2min}]^{Rd}$, was prepared by immersing the resting enzyme in D_2O buffer for 2 minutes prior to its re-reduction to the ferrous state for the spectral measurements. The difference spectra calculated by using $[CcO^{Ox}{}_{D}]^{Rd}$ as a reference are shown in Figure S6 (Spectrum c and b, respectively)**.** The data show that the two difference spectra are almost identical, demonstrating that the H/D exchange in the pulsed enzyme is the same as that in the resting enzyme.

6) *The role of water channels in proton translocation*.

The H-channel is composed of a series successive water accessible cavities leading from the **n**-side surface of the membrane to the vicinity of the heme *a* [5,6]. Combining the crystal structure data, in which the cavity sizes in the oxidized state of the enzyme are reduced [6], with site-directed mutational studies [5], a model was proposed in which water passage through the cavities is allowed in the reduced state of the enzyme and prohibited in the oxidized state [7]. It was postulated that protons are then translocated from the water pockets along heme *a* to D51 on the **p**-side of the membrane controlled by the heme *a* oxidation state and the heme a_3 ligand binding states. In that model, the gate was ascribed to the presence of pockets that could translocate water molecules in the reduced structure but not in the oxidized structure. The gate observed in this study is, however, not assignable to the gate in the water pockets, as the H/D exchange data demonstrate that the gate is closed in the reduced state and open in the

oxidized state, opposite to the relative expansion and contraction of the water channel. However, the presence of the water channel is important for the model described here as it allows for proton translocation via a Grotthus mechanism [8].

References

1. Nicholls P, Petersen LC, Miller M, Hansen FB (1976) Ligand-induced spectral changes in cytochrome c oxidase and their possible significance. Biochim Biophys Acta 449: 188-196.
2. Ji H, Yeh SR, Rousseau DL (2005) Structural Characterization of the $P_{CO/O2}$Compound of Cytochrome c Oxidase. FEBS Lett 579: 6361-6364.
3. Kim Y, Shinzawa-Itoh K, Yoshikawa S, Kitagawa T (2001) Presence of the Heme-oxo Intermediate in Oxygenation of Carbon Monoxide by Cytochrome *c* Oxidase Revealed by Resonance Raman Spectroscopy. J Am Chem Soc 123: 757-758.
4. Malatesta F, Antonini G, Sarti P, Brunori M (1995) Structure and Function of a Molecular Machine: Cytochrome *c* Oxidase. Biophys Chem 54: 1-33.
5. Shimokata K, Katayama Y, Murayama H, Suematsu M, Tsukihara T, et al. (2007) The proton pumping pathway of bovine heart cytochrome c oxidase. Proc Natl Acad Sci U S A 104: 4200-4205.
6. Tsukihara T, Shimokata K, Katayama Y, SHimada H, Muramoto K, et al. (2003) The Low-Spin Heme of Cytochrome *c* Oxidase as the Driving Element of the Proton-Pumping Process. Proc Natl Acad Sci U S A 100: 15304-15309.
7. Muramoto K, Ohta K, Shinzawa-Itoh K, Kanda K, Taniguchi M, et al. (2010) Bovine cytochrome c oxidase structures enable O2 reduction with minimization of reactive oxygens and provide a proton-pumping gate. Proc Natl Acad Sci U S A 107: 7740-7745.
8. Belevich I, Verkhovsky MI (2008) Molecular mechanism of proton translocation by cytochrome c oxidase. Antioxid Redox Signal 10: 1-29.
